# Pre- and Postoperative Voice Therapy for Benign Vocal Fold Lesions: An International Electronic Delphi Consensus Study

**DOI:** 10.1016/j.jvoice.2022.12.008

**Published:** 2025-05

**Authors:** Anna White, Paul Carding, Vicky Booth, Pip Logan, Julian McGlashan, Rehab Awad

**Affiliations:** ⁎ Centre for Rehabilitation & Ageing Research, Academic Unit of Injury, Recovery and Inflammation Sciences, School of Medicine, University of Nottingham, Nottingham, UK; † Oxford Institute of Midwifery, Nursing and Allied Health Research, Oxford, UK; ‡ Nottingham University Hospitals NHS Trust, Nottingham, UK; § University Hospital Lewisham NHS Trust, London, UK; # Kasr Al-Aini Hospital, Cairo University, Cairo, Egypt

**Keywords:** Pre- and postoperative, Benign vocal fold lesions, Voice therapy, Phonosurgery, Delphi, Consensus

## Abstract

**Introduction:**

Voice therapy management of benign vocal fold lesions (BVFLs) is variable and there are currently no clinical guidelines. Poor descriptions of voice therapy interventions lead to unwarranted variation in treatment. Triangulation of the current evidence identifies a number of potential best practice elements, but also a number of outstanding questions to be explored. The aim of this study was to refine and gain global consensus on “best practice” for a pre- and postoperative voice therapy intervention for adults with BVFLs.

**Methods:**

An international sample of expert voice therapists (*n* = 42) were recruited to take part in this three-round electronic modified Delphi study. Participants were presented with statements concerning a pre- and postoperative voice therapy intervention. Statements were developed from previous research and based on the TIDieR checklist (eg, why, when, what, how?) Participants rated the extent to which they agreed or disagreed with a statement and gave comments to support their response. Consensus was defined as >75% of participants agreeing or strongly agreeing with a given statement. If consensus was not reached, participant comments were used to generate new statements and were rated in the next round. Stability of consensus between rounds was assessed.

**Results:**

The 42 international experts achieved consensus on 33 statements relating to components of a best practice pre- and postoperative voice therapy intervention for patients with BVFLs. Consensus on statements ranged from 81% to 100%. These statements were explicitly mapped to the TIDieR checklist to ensure that all aspects of the intervention were considered and the questions of “why, what, how, when and individual tailoring” were addressed.

**Conclusions:**

This study has significantly enhanced our understanding of what should be in a best practice pre- and postoperative voice therapy intervention. It is important to now test these findings for acceptability and feasibility, prior to considering effectiveness research.

## INTRODUCTION

Benign vocal fold lesions (BVFLs), such as vocal fold cysts and polyps account for between 22% and 68% of those seeking help for dysphonia.^1^ These lesions can cause dysphonia in various ways depending on the lesion type, position, and size, and the individual's voice requirements and ability to compensate for the lesion presence. BVFLs may prevent full vocal fold closure, impact on vibratory characteristics, and increase the likelihood of compensatory muscle tension.^2^^,^^3^ This can lead to changes in the acoustic and perceptual qualities of the voice, as well as impacting vocal function, and increasing vocal tract discomfort for some individuals.

Patients with BVFLs may be offered surgery, voice therapy or pharmacological management.^4^ Phonosurgery can improve both voice quality and voice related quality of life,^5^, ^6^, ^7^, ^8^, ^9^ however surgery is costly,^10^ has associated risks^11^ and is not always required.^12^, ^13^, ^14^ There is some evidence that voice therapy delivered in addition to phonosurgery may improve both voice and quality of life outcomes.^15^^,^^16^ However, there is no clear understanding of what constitutes voice therapy for this population. A recent Systematic Review of the components of pre- and postoperative voice therapy found that information was frequently lacking regarding the content, timing and intensity of the reported voice therapy intervention^17^ in studies reporting voice outcomes following surgery. It found that voice therapy content was sometimes described according to the specific technique used (eg, Accent method^18^) but more frequently it was described according to a broad aim of the intervention (eg, Breath support). An interview study with expert clinicians highlighted numerous factors which influence pre- and postoperative voice therapy (PaPOV),^19^ and a survey of current practice showed high levels of variability between clinicians, fuelled by a lack of guidance and evidence.^20^ Without a clear understanding of what best practice may look like, and in time, evidence to support the effectiveness of a detailed intervention, patients risk receiving suboptimal care,^21^ additional medical appointments, prolongation of symptoms,^9^ suffer complications from surgery,^22^ raised anxiety and financial consequences.^23^

Voice therapy is a highly complex intervention involving multiple interacting elements. Voice therapy typically comprises both direct (exercises) and indirect (information and advice) interventions. Voice exercises may focus on one or all of the three subsets of voicing; breathing, phonation and resonance. Clinicians use many different techniques, sometimes concurrently, to achieve improved vocal quality. Unique complexities exist for patients with dysphonia who are due to undergo surgery to remove BVFLs. Clinicians must help patients prepare for surgery and consider wound healing, re-establishing the mucosal wave, postoperative vocal rehabilitation and avoid lesion recurrence by tackling causative factors. Developing complex interventions involves multiple phases, each impacting, informing and directing the next.^24^ Guidance from the Medical Research Council (MRC) emphasizes the importance of examining the best available evidence and undertaking appropriate theory and modeling prior to pilot and feasibility testing.^25^^,^^26^

There is a substantial theoretical basis for PaPOV based on models of wound healing,^27^, ^28^, ^29^, ^30^ prehabilitation,^31^ motor learning,^32^, ^33^, ^34^ and behavior change,^35^, ^36^, ^37^ but their application to this population has not been fully explored. Triangulation of the current evidence^38^ found stability of consensus among data sources to support the inclusion of 29 out of 61 potential “components” or “ingredients” in a “best practice” PaPOV intervention. Findings of this triangulation study supported the role for voice therapy both pre- and postoperatively. It highlighted the importance of information provision in the preoperative phase and the need to address factors which may have contributed to lesion development such as phonotrauma, exposure to irritants and unhelpful muscle tension patterns. It also highlighted the need for further work to explore a number of outstanding questions.

The aim of this Delphi study was to refine and gain global consensus on perceived “best practice” for a pre- and postoperative voice therapy intervention (PaPOV) for adults with BVFLs. The objectives were to reach consensus on components of a best practice intervention, to explore and expose diverse views among expert clinicians, and to progress new knowledge. The outcome of which would be the development of a robust, detailed intervention protocol (PaPOV) based on the TIDieR checklist^39^ which could be used in a future feasibility trial.

## METHODS

### Study design

This study used a three round electronic modified Delphi methodology^40^ with an international expert sample. The Delphi technique uses multistage questionnaires and samples experts on the study topic, to obtain consensus on an issue where no agreement previously exists.^40^^,^^41^ Delphi methodology has been widely and successfully used in health research, as a cost and time effective consensus method. The Delphi technique does not produce right or wrong answers, rather it leads to the generation of valid consensus in expert opinion. Participant anonymity allows increased freedom of expression and contribution. Feedback between rounds allows participants to consider the group's response in relation to their own.

### Expert panel recruitment

#### Panelists

An international sample of experts in the field, recruited purposively, were invited to take part. Delphi sample size recommendations depend on the complexity of the topic under investigation and participant homogeneity.^40^ Attrition is reported to be higher if participants lack sufficient interest in the topic. Therefore, the sampling strategy and size gave these factors due consideration. Participants’ expertise was judged on their clinical, professional, and academic background, together with BVFL experience. Participants were required to have a professional qualification permitting them to practice as a Speech and Language Therapist, Speech and Language Pathologist, Logopedist, or Phoniatrician, depending on their country of work. Participants needed both a specified level of experience (>5 years’ experience working with BVFLs, >50% time working in voice disorders, >2 patients per month with BVFLs) and a self-reported interest in the topic. A sample of 40 participants was anticipated to result in a final round sample of approximately 36 clinicians (predicted 10% attrition).^42^ This was considered appropriate to generate sufficient data without diluting participant expertise. A target was set for over 30% of participants to be recruited from centers outside the authors’ country to ensure that there was international representation.

#### Recruitment

Participants were approached via:

• Professional networks (eg, RCSLT, Voice Clinical Excellence Networks, British Laryngological Association)
• Social media (eg, Twitter using international networks and contacts)
• Snowballing through the authors’ networks (eg, Australia, America, Germany, Egypt)

Participant information sheets and inclusion criteria were distributed by e-mail. If a potential participant confirmed that they met the criteria and would like to be involved, their details were kept and a link to the questionnaire was sent on the day of the launch. Consent was gained electronically at the point of questionnaire participation. Data was collected regarding the time taken to respond, the response rate at each round, and where available, reasons for attrition.

### Survey development

Delphi Statements were derived from preliminary intervention development work.^17^^,^^19^^,^^43^ The intervention was initially described according to the TIDieR Checklist^39^ and 61 statements which contained “targets” and “ingredients”^44^ of the intervention were detailed. A triangulation of the current evidence^38^ identified where there was sufficient evidence for the inclusion of the statement into the intervention, and highlighted where outstanding questions, inadequate exploration, or disagreement in the current evidence base existed. These statements, together with a few introductory statements, formed round one of the Delphi study.

**FIGURE 1:**
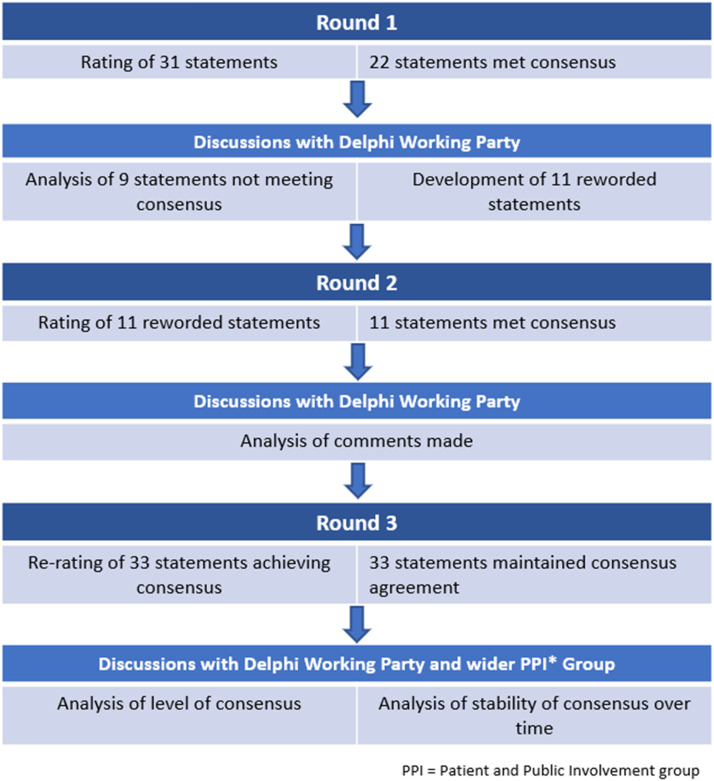
Flow diagram illustrating the three-round Delphi process.

The questionnaire was developed using the JISC online surveys platform (Jisc©, 2022). The questionnaire was piloted by ten individuals (including patients *n* = 2, researchers *n* = 2, clinical academics *n* = 3, and clinicians *n* = 3) to gain feedback regarding visual appearance, functionality, linguistic and semantic content. Modifications were made based on the feedback received. A Delphi working party (DWP) reviewed the final statements prior to the launch. The DWP included a Voice therapist (RA), a Laryngologist (JM), three patients with lived experience of BVFLs (DB, DS, CE) and an independent researcher (PP) experienced in Delphi methodology. The DWP reviewed findings, summaries of comments and the newly generated statements between rounds. They ensured that new statements reflected participants’ comments. Additional expertise was sought between rounds 1 and 2 from a clinician (JB) with expertise in behavior change techniques (BCT) relevant to voice therapy, to ensure that one new statement was in line with broader BCT literature. Figure 1 illustrates the three-round design.

#### Round 1

Participants were asked to rate a series of statements using a five-point Likert scale (Strongly Disagree, Disagree, Neither Agree nor Disagree, Agree, Strongly Agree) on the extent to which they felt the statement represented an integral part of a best practice PaPOV intervention. Participants were asked to base their response on their knowledge of the evidence, clinical observations and beliefs about “best practice.” They were reminded that this may or may not represent “current practice” in their institution. The use of a five point Likert scale sat within the suggested optimal number of proposed categories.^4^, ^5^, ^6^, ^7^^,^^42^ For each statement, a comments box permitted further justification of responses or allowed the participant to seek clarification.

Participant demographics were gathered in round one. These included gender, age, country of work, number of years of practice, % of clinical caseload in voice and highest educational qualification. Participants were asked to complete the questionnaire within 2 weeks, with email reminders being sent on days 5 and 10. After 2 weeks, any participant who had not yet completed the questionnaire was emailed and given a 3-day extension. Failure to respond in this time frame was regarded as participant attrition and no further communication was sent.

#### Round 2

Participants were sent a summary of responses from round one, including percentages of responses to each statement and a summary of the comments made for any statement which did not achieve consensus. Reworded statements, based on participants’ feedback and comments were included for re-evaluation, using the process described in round one.

#### Round 3

Participants were sent a final set of statements to rate. This included all statements which received consensus in previous rounds. By asking participants to re-rate statements from earlier rounds, a judgment on the stability of consensus over time could be formed. This is an important quality indicator in Delphi methodology.^42^

### Ethics

This study was reviewed by the University of Nottingham, Faculty of Medicine and Health Sciences Research Ethics Committee on October 5th, 2021 and given a favorable ethics opinion (FMHS 260-1021).

Ethical responsibilities toward the expert sample and research community were considered throughout the conception and delivery of the study. Participants received a full written explanation of the study, their involvement and the level of anonymity afforded to them. They were given contact information for any questions, comments or concerns. Email reminders were sent in accordance with the recruitment strategy and wording was carefully reviewed to reduce any risk of participant coercion. Participants’ identities were not disclosed and any potentially identifiable information in comments was removed before sharing with participants.

### Data analysis

Descriptive statistics were used to describe participants’ demographic characteristics and group responses to each statement. Consensus was defined *a priori* as >75% of participants rating a statement as “Agree” or “Strongly Agree”; consistent with a review of Delphi study methodology.^42^

Comments for all statements were reviewed and key words and themes were highlighted. Comments were used to reword any statement which did not reach consensus. Analysis of the qualitative data was undertaken by two researchers, AW and PC, with further input from the study team (VB and PL). DWP members reviewed findings from each round to ensure intervention acceptability, theoretical grounding and ensure that participants’ comments were adequately considered.

The stability of the consensus between two rounds was judged by comparing the percentage of agreement between the first and second time a statement was presented, the mean response and the standard deviation. The percentage agreement showed whether the overall consensus had increased or decreased between rounds. Comparison of the standard deviation showed whether the groups’ response had a greater or lesser variation for each statement when rated for a second time.

## RESULTS

### Demographics

In total, 45 participants from 15 countries participated in round one. Of these, 44 participants completed round two, and 42 completed round three. Table 1 presents the participants demographics for each round. Participants had a high degree of expertise and spent a considerable proportion of their clinical practice working with patients with voice disorders. A gender bias toward female participants was consistent with employment figures in the Speech and Language Therapy profession.^45^

**TABLE 1:** Participant Demographics

	Round 1		Round 2		Round 3	
Number of participants	45		44		42	
Number of countries	15		14		13	

	*N*	%	*N*	%	*N*	%
International representation (outside UK)	20	44%	20	45%	18	43%
Years’ Experience (*n*, and %)						
5-9	7	16%	7	16%	7	17%
10-19	20	44%	20	45%	20	48%
20-29	14	31%	13*	30%	13	31%
30-40	3	6%	3	7%	2*	5%
Over 40 years	1	2%	1	2%	0*	0%
% of time working in voice disorders						
50-75%	19	42%	19	43%	19	45%
>75%	26	58%	25*	57%	23*	55%
Age (*n*, and %)						
20-30 years	4		4		4	
31-40 years	14		14		14	
41-50 years	17		16*		16	
51-60 years	8		8		7*	
61-70 years	1		1		0*	
Prefer not to say	1		1		1	
Gender						
Female	40	89%	39*	89%	37*	88%
Male	5	11%	5	11%	5	12%

⁎ Denotes participant attrition.

### Summary of intervention (according to TIDieR domains)

Table 2 summarizes the number of Delphi statements for each TIDieR domain^39^ across the three rounds. In round one, all statements relating to the underlying theory and rationale (*Why*), therapeutic skills (*How*) and individual variation (*Tailoring*) met consensus (>75% agree or strongly agree). However, there was variation in the levels of consensus across other domains. Only a third of statements relating to dosing, timing, and frequency (*When and How much*) achieved consensus. Two statements relating to the materials provided to patients, and five relating to procedures, (*What*) also failed to reach the preset consensus criteria in round one.

**TABLE 2:** Delphi Statement Representation on the TIDieR Checklist for Each Round

Domain Based on TIDieR Checklist	Number of Statements in Each Domain			Proportion of Statements Where Consensus was Achieved (*n*)		
	Round 1	Round 2	Round 3	Round 1	Round 2	Round 3
*Why*: Describes any rationale, theory, or goal of the elements essential to the intervention	3	0	3	100% (3)	N/A	100% (3)
*What*: Materials: Describes any physical or informational materials used in the intervention, including those provided to participants or used in intervention delivery or training	6	2	6	66.7% (4)	100% (2)	100% (6)
*What*: Procedures: Describes each of the procedures, activities and/or processes used in the intervention	13	6	15	61.5% (8)	100% (6)	100% (14)
*How*: Describes the modes of delivery of the intervention and whether it was provided individually or in a group. Includes therapeutic skills and techniques of the clinician.	4	0 (3)*	4	100% (4)	N/A	100% (4)
*When and How much*: Describes the number of times the intervention as delivered and over what people of time including the number of sessions, their schedule and their duration, intensity or dose.	3	3	6	33.3% (1)	100%(3)	100% (6)
*Tailoring*: If the intervention is planned to be personalized, titrated or adapted, this describes what, why, when and how.	2	0 (10)*	2	100% (2)	N/A	100% (2)
Total	31	11	33	71% (22)	100% (11)	100% (33)

⁎ Most statements in round 2 addressed two or three TIDieR checklist domains in one statement, as a result of increased detail or clarification. For example, “What” plus “tailoring.” Secondary domains are shown in brackets.

### Round one consensus

**FIGURE 2:**
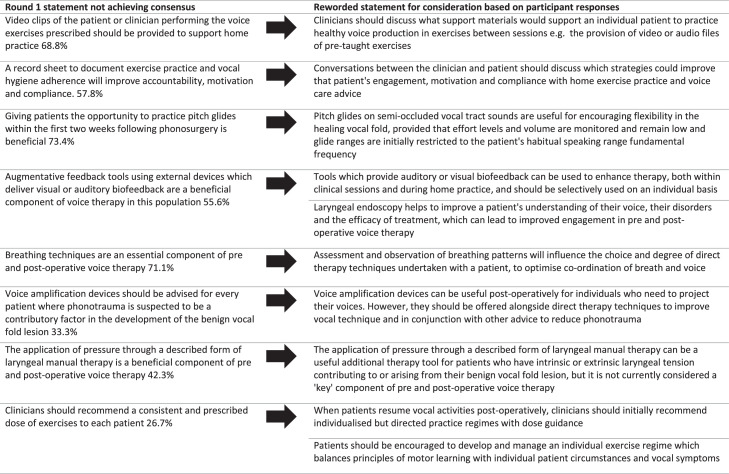
Rewording of statements not achieving consensus in round 1.

Participants rated 31 statements in round one. Appendix A presents the groups’ responses, showing the mean and standard deviation (SD) for each statement, the percentage of participants rating a statement as “Agree” or “Strongly Agree” (termed consensus agreement %) and a summary of the participants’ comments. Of these 31 statements, 22 statements met consensus, which ranged from 75.5% to 100%. Nine statements failed to reach consensus (level of agreement 11.1-71.1%). These statements were revised and refined for further consideration in round two and are listed in Figure 2.

Participants agreed on the underlying principles of the intervention: The intervention should be offered both pre- and postoperatively, there should be a balance between wound healing and remobilization of the vocal fold epithelium, and providing opportunities for regular practice and functional tasks would contribute to learning new vocal skills. Participants agreed that clinicians should deliver information in a way that would maximize engagement and adherence, and continuous clinician assessment of the patients’ presentation and performance would inform the pace and direction of hierarchical tasks.

In terms of the materials provided, participants agreed that patients should be given individualized recommendations to supplement generic advice, with information being provided in multiple formats (eg, written and verbal). Preoperative information should enable a participant to improve voice care and tackle specific factors which may have contributed to lesion development. Postoperative advice should include specific examples of graded vocal tasks at different time points.

Consensus was achieved regarding the role of patient discrimination. Participants agreed that improving patients’ discrimination of concepts relating to volume, voice quality and resonance was essential and could help to improve self-efficacy, self-monitoring and adherence. There was agreement that a short period of absolute voice rest should be followed by relative voice rest, including the use of confidential voice, and that gentle vocalization should recommence within the first week. Postoperative intervention should include the use of semioccluded vocal tract exercises to reduce collision forces at the glottis, and in time, the opportunity to practice projection techniques may be beneficial.

With regard to the dosing of exercises, it was felt that clinicians and patients should agree an individual dose of exercises which aligned with principles of muscle memory and habit formation. All participants agreed that the number of voice therapy sessions should be tailored to the patient's vocal and psychological needs, style of learning, and motivation.

### Round two consensus

In round two there was a 98% response rate. Participants were presented with 11 new statements developed from the nine which failed to reach a consensus in round one. New statements incorporated participants’ wording where appropriate and are shown in Figure 2. Some statements related to “optional” or “additional” components which could be included on a case-by-case basis rather than being essential to the intervention. These included laryngeal manual therapy, breath support work and the use of amplification devices.

All statements in round two met consensus, defined as >75% of participants agreeing or strongly agreeing with the statement. Appendix B presents the full groups’ responses, showing the mean and standard deviation (SD) for each statement, the percentage of participants rating a statement as agree or strongly agree (termed consensus agreement %) and a summary of the participants’ comments.

### Round three consensus

The response rate for round three was 95% (*n* = 42). Participants re-rated 33 statements which had gained consensus in either round one or two. All statements maintained consensus. Table 3 shows the percentage of consensus agreement, mean and SD for all 33 statements in the final Delphi round. Appendix C provides a summary of round three comments.

**TABLE 3:** Statements Achieving Consensus in Round Three in Order of Highest % Agreement

Round 3 Statement	Mean (SD)	Consensus Agreement
A preoperative checklist should include advice regarding voice care, voice conservation, reflux management, and communication strategies for use during postoperative voice rest.	4.50 (0.506)	100%
Clinicians should use multimodality feedback techniques to enhance a patient's learning and progression in exercises.	4.93 (0.261)	100%
Dosing recommendations should optimize muscle memory and habit formation.	4.81 (0.397)	100%
Patients who are undergoing phonosurgery for benign vocal fold lesions should be offered pre- and postoperative voice therapy.	4.81 (0.671)	97.60%
Providing opportunities to practice appropriate voice use through regular exercises and functional tasks will contribute to learning new vocal skills.	4.74 (0.701)	97.60%
Information should be available to patients in multiple modalities where possible.	4.69 (0.715)	97.60%
Clinicians should discuss what materials would support an individual patient to practice healthy voice production in exercises between sessions, eg, the provision of video or audio files of pretaught exercises	4.60 (0.734)	97.60%
Conversations between the clinician and patient should discuss which strategies could improve that patient's engagement, motivation and compliance with home exercise practice and voice care advice.	4.62 (0.731)	97.60%
Patients should be encouraged to resume gentle vocalization within the first week following phonosurgery.	4.71 (0.508)	97.60%
Semioccluded vocal tract (SOVT) exercises using an anatomical structure or external vehicle are a beneficial component of voice therapy in this population.	4.64 (0.533)	97.60%
Laryngeal endoscopy helps to improve a patient's understanding of their voice, their disorder, and the efficacy of treatment, which can lead to improved engagement in pre- and postoperative voice therapy.	4.55 (0.550)	97.60%
Clinicians should use a range of strategies to deliver information in a way which maximizes patient engagement and adherence in therapy.	4.83 (0.660)	97.60%
Continuous clinician assessment of the patient's presentation and performance will inform the pace and direction of hierarchical tasks.	4.86 (0.647)	97.60%
The number of voice therapy sessions should be tailored to the patient's vocal and psychological needs, style of learning, and motivation.	4.83 (0.660)	97.60%
Following phonosurgery, a balance must be achieved between principles of voice rest (wound healing) and remobilization of the epithelium.	4.67 (0.902)	95.20%
The intensity of clinician directed feedback to the patient will be reduced as the patient's self-evaluation accuracy improves.	4.64 (0.759)	95.20%
When patients resume vocal activities postoperatively, clinicians should initially recommend individualized but directed practice regimes with dose guidance.	4.14 (0.472)	95.20%
Clinicians and patients should agree an individually tailored dose of exercises.	4.64 (0.727)	95.20%
Written postoperative voice use guidance should include graded tasks with examples of voice use at different time points in rehabilitation.	4.21 (0.645)	92.90%
Developing the discrimination skills to detect and monitor changes in voice quality is an essential component of voice therapy in this population.	4.43 (0.630)	92.90%
A period of relative voice rest should be recommended following phonosurgery.	4.50 (0.944)	92.90%
Pitch glides on semioccluded vocal tract sounds are useful for encouraging flexibility in the healing vocal fold, provided that effort levels and volume are monitored and remain low and glide ranges are initially restricted	4.57 (0.630)	92.90%
Giving patients the opportunity to practice increased levels of muscle activation during vocalization in exercises and speech tasks postoperatively is beneficial.	4.19 (0.552)	92.90%
Tools which provide auditory or visual biofeedback can be used to enhance therapy, both within clinical sessions and during home practice, and should be selectively used on an individual basis.	4.19 (0.740)	92.90%
Assessment and observation of breathing patterns will influence the choice and degree of direct therapy techniques undertaken with a patient, to optimize co-ordination of breath and voice.	4.52 (0.634)	92.90%
Voice amplification devices can be useful postoperatively for individuals who need increased volume output. However, they should be offered alongside direct therapy techniques to improve vocal technique, and in conjunction with advice to reduce phonotrauma.	4.31 (0.604)	92.90%
Patients should be encouraged to develop and manage an individual exercise regime which balances principles of motor learning with individual patient circumstances	4.60 (0.767)	90.50%
Developing the discrimination skills to be able to detect and monitor volume changes in the voice is an essential component of voice therapy in this population.	3.95 (0.661)	88.10%
Developing the skills to be able to detect and monitor changes in vocal tract resonance is an essential component of voice therapy in this population.	4.12 (0.832)	88.10%
The application of pressure through a described form of laryngeal manual therapy (LMT) can be a useful additional therapy tool for patients who have intrinsic or extrinsic laryngeal tension contributing to or arising from their BVFL, but it is not currently considered a “key” component of pre- and postoperative voice therapy.	4.07 (0.640)	88.10%
A minimum number of 1 pre- and 1 postoperative voice therapy sessions could be recommended as a guide for anyone undergoing phonosurgery, with the option to increase this according to patient and surgical factors.	4.17 (1.167)	83.30%
A personalized goal setting sheet should supplement generic advice sheets, to optimize compliance by identifying barriers and facilitators relevant to the patient's situation.	4.14 (0.843)	81%
A period of absolute voice rest, including avoidance of all laryngeal valving activities should be recommended following phonosurgery.	4.00 (0.988)	81%

**FIGURE 3:**
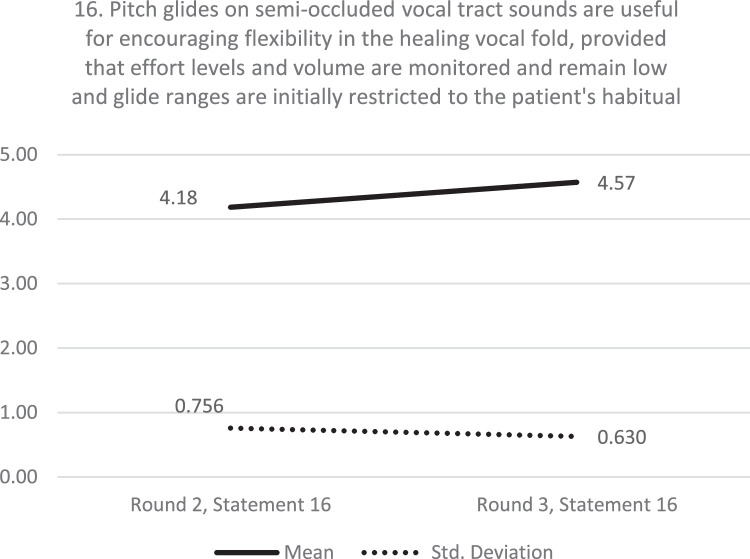
Stability of consensus between rounds showing typical pattern of increased mean response and decrease variation in the group response between time point 1 and 2.

The percentage of participants “agreeing” or “strongly agreeing” with a statement increased between rounds for 21 of the statements, decreased for 10 statements and was unchanged at 100% for two statements. The overall mean was 4.44 and 4.49 for the first and second presentation of statements respectively. This illustrates a high level of group stability and a general shift toward greater levels of consensus as the Delphi study progressed. Figure 3 shows a graphical representation of the stability of consensus for a statement relating to pitch glides with the full data included in the supplementary material, Appendix D.

## DISCUSSION

This three-round modified Delphi study achieved its aims and objectives by reaching global consensus, from a panel of 42 international experts on 33 statements relating to the components of a best practice pre- and postoperative voice therapy intervention (PaPOV) for patients with benign vocal fold lesions.

### The PaPOV intervention

Statements were explicitly mapped to the TIDieR checklist to ensure that all aspects of the intervention were considered and the questions of “why, what, how, when and individual tailoring” were addressed. This follows the suggested guidance for improving the quality of intervention descriptions allowing clinicians and researchers to replicate and build upon research findings.^39^ The wording of statements in all rounds aligned with the principles of Treatment Theory outlined described in the Rehabilitation Treatment Specification System (RTSS) for voice therapy.^46^ Considerable work has been undertaken to identify unique ingredients in voice therapy.^46^, ^47^, ^48^ Use of the RTSS as a framework to describe the intervention, aimed to reduce the instance of multiple components being included which were likely to be different in language, but similar in action. For example, rather than include a statement about patients being taught specific voice therapy techniques, for example, Lax Vox, Straw phonation, Lip Trills, Kazoo, or Accent Method, one statement referred to the opportunity to practice semioccluded vocal tract exercises. The RTSS emphasizes the importance of describing the type of dosing (amount of practice), feedback, and progression rules of any ingredient. Statements specifically sought participants’ views on these aspects of the intervention. However, given that Delphi methodology emphasizes the importance of using participants’ own language in rephrased statements,^42^ the statements did not use RTSS terminology verbatim.

The stability of the groups’ response was high. Small increases in the groups’ response over time were predominantly noted and may be explained by participants’ reflections on their own response in light of the whole group response.^40^ In this study, participants shared comments, justifications, posed questions and referred to published literature on related topics. This is anticipated to have deepened understanding and shaped participants’ views in subsequent rounds.

There was strong consensus in statements which related to the rationale and theoretical underpinnings of the PaPOV intervention. Participants agreed that motor learning and exercise physiology principles^49^, ^50^, ^51^ underpinned the intervention and supported opportunities for regular practice both in exercises and in functional tasks. Dosing recommendations which optimized habit formation would help patients to learn new vocal skills, which in turn would improve long-term self-management and prevention of lesion recurrence. This is consistent with the broader literature on voice therapy interventions.

Participants recognized the need to balance principles of wound healing following phonosurgery with remobilization and felt this distinguished PaPOV interventions from other voice therapy interventions. There was consensus that, “*A period of absolute voice rest*,” should be followed by “*a period of relative voice rest*” and that, “*Patients should be encouraged to* ***resume*** *gentle vocalization within the first week following phonosurgery*.” Studies investigating the impact of voice rest on voice outcomes following phonosurgery report varied findings.^52^, ^53^, ^54^, ^55^ Kaneko et al^52^ reported statistically significant improvement in acoustic, perceptual and patient reported outcomes in those who adhered to 3 days of voice rest compared to 7 days. However, others have shown no significant differences in voice outcomes when varying postoperative voice rest protocols.^53^^,^^55^^,^^56^ Patients find compliance to prolonged periods of absolute voice rest difficult^55^ and there is evidence that early mobilization may initiate positive biological changes associated with improved function.^57^^,^^58^ Despite this, some centers recommend prolonged periods of voice rest, with positive voice outcomes following phonosurgery.^59^^,^^60^ In the midst of dissonance between clinical and laboratory research, and current clinical practice, participants have taken a pragmatic balance, which draws on the current evidence base and considers patients’ needs.

Voice therapy is a behavior change intervention, requiring the engagement and active participation of patients.^61^ Although behavior change has been studied extensively in some health settings such as weight loss^62^ and smoking cessation,^63^ the application of behavior change techniques (BCT) to patients with voice disorders is less frequently studied. Participants agreed that the PaPOV intervention was underpinned by BCT, and statements highlighted the practical applications of this which included improving a patient's capability, opportunities and motivation to change a behavior through the provision of individualized, tailored interventions and joint goal setting.

### Strengths and limitations

Gaining consensus in 33 statements reflecting aspects of the TIDieR framework has resulted in a comprehensive description of the PaPOV intervention.

Delphi has no single methodology and has been criticized for its lack of methodology. Therefore care was taken to explicitly describe methodological choices taken within this study. Several steps have improved the quality of the work, including piloting questionnaires with researchers, clinicians and PPI, in-depth interrogation of participant comments, the involvement of a multidisciplinary Delphi Working Party and an analysis of consensus stability.^40^, ^41^, ^42^ The content validity of the Delphi statements was enhanced in three ways. First, by using preliminary intervention development work to highlight outstanding questions, second, by ensuring that the panel was a representative sample of relevant experts who were motivated to participate^41^^,^^64^ and third by giving participants opportunities to comment on the statements presented^65^ thereby giving greater depth and justification to their decisions.

This Delphi study recruited participants from 15 countries. The international sample recruited broadened the generalizability of study findings. While a range of sampling techniques were used to identify appropriate participants and advertise the study, it is certain that individuals with significant “expertise” did not take part and would have important contributions to offer. It is important to remember that the results of the Delphi study are specific to the panel of experts sampled^40^ and that consensus does not mean that the “correct” answer has been found. It is acknowledged that the Delphi technique is not a substitute for other forms of evidence. Rather, this study and the knowledge gained has facilitated further research to be undertaken.

### Conclusions and implications for current practice

Expert clinicians have reached an agreement on 33 statements regarding a best practice Pre- and Postoperative Voice therapy intervention (PaPOV) for patients with benign vocal fold lesions. There is consensus among experienced voice clinicians that PaPOV is underpinned by wound healing, exercise physiology and behavior change. A number of components are considered essential in PaPOV, and others are considered additional, based on individual assessment and interpretation of the patient's presentation. Preoperative involvement is considered fundamental and can be used as an opportunity to improve patient understanding, engagement in the therapeutic process and set joint goals. The importance of tackling factors which have contributed to lesion development through direct and indirect activities is recognized. Clinicians also consider developing patients’ discriminatory skills and providing opportunities to practice efficient styles of voicing, which is essential for long term vocal skill development.

This Delphi study has enabled consensus to be obtained regarding expert clinicians’ view of best practice management of BVFLs for individuals undergoing phonosurgery. It immediately offers practicing clinicians guidance relating to all aspects of intervention which can be considered with this patient population. The Medical Research Council's framework for Developing and Evaluating complex interventions^26^ emphasizes the importance of preliminary intervention development work to explore the theoretical underpinnings of the intervention, explore key uncertainties, engage stakeholders and refine the intervention. The findings from this and other intervention development work^38^ have been used to develop an intervention protocol, ready for feasibility testing.

## Appendix SUPPLEMENTARY DATA

Image, application 1

Image, application 2

Image, application 3

Image, application 4

## References

[1] McGlashan J, Costello D, Bradley P, Ludman H, Bradley P (2007). ABC of Ear, Nose and Throat.

[2] Carding P. (2017).

[3] Toran K, Lal B. (2010). Objective voice analysis for vocal polyps following microlaryngeal phonosurgery. Kathmandu Univ Med J.

[4] Sulica L, Behrman A. (2003). Management of benign vocal fold lesions: a survey of current opinion and practice. Ann Otol Rhinol Laryngol..

[5] Cho KJ, Nam IC, Hwang YS (2011). Analysis of factors influencing voice quality and therapeutic approaches in vocal polyp patients. Eur Arch Oto-Rhino-Laryngol.

[6] Jensen JB, Rasmussen N. (2013). Phonosurgery of vocal fold polyps, cysts and nodules is beneficial. Danish Med J.

[7] Akbulut S, Gartner-Schmidt JL, Gillespie AI (2016). Voice outcomes following treatment of benign midmembranous vocal fold lesions using a nomenclature paradigm. Laryngoscope.

[8] Catani G, Hamerschmidt R, Moreira A (2016). Subjective and objective analyses of voice improvement after phonosurgery in professional voice users. Sci Med.

[9] Lee YS, Lee DH, Jeong G-E (2017). Treatment efficacy of voice therapy for vocal fold polyps and factors predictive of its ffficacy. J Voice.

[10] Cohen SM, Kim J, Roy N (2012). Direct health care costs of laryngeal diseases and disorders. Laryngoscope.

[11] Allen J. (2010). Causes of vocal fold scar. Curr Opin Otolaryngol Head Neck Surg.

[12] Schindler A, Mozzanica F, Ginocchio D (2012). Vocal improvement after voice therapy in the treatment of benign vocal fold lesions. Acta Otorhinolaryngol Ital.

[13] Jeong W-J, Lee SJ, Lee WY (2014). Conservative management for vocal fold polyps. JAMA Otolaryngol–Head Neck Surg..

[14] Zhuge P, You H, Wang H (2016). An analysis of the effects of voice therapy on patients with early vocal fold polyps. J Voice.

[15] Tang SS, Thibeault SL. (2017). Timing of voice therapy: a primary investigation of voice outcomes for surgical benign vocal fold lesion patients. J Voice.

[16] Ju Y, Jung KY, Kwon SY (2013). Effect of voice therapy after phonomicrosurgery for vocal polyps: a prospective, historically controlled, clinical study. J Laryngol Otol.

[17] White AC, Awad R, Carding P. Pre and Post-operative Voice Therapy Intervention for Benign Vocal Fold Lesions: A Systematic Review. J Voice. 2021:S0892-1997(21)00191-0. 10.1016/j.jvoice.2021.06.005. Epub ahead of print. PMID: 34272141. 34272141

[18] Thyme-Frokaer K. (2001). The Accent Method: A Rational Voice Therapy in Theory and Practice.

[19] White AC, Carding P. (2020). Pre- and postoperative voice therapy for benign vocal fold lesions: factors influencing a complex intervention. J Voice.

[20] White A. Pre and Post-operative voice therapy: a UK survey of current practice. 2020 (In Press).

[21] Benninger MS. (2011). Quality of the voice literature: what is there and what is missing. J Voice.

[22] Friedrich G, Dikkers F, Arens C (2013). Vocal fold scars: current concepts and future directions. Consensus report of the phonosurgery committee of the European laryngological society. Eur Arch Otorhinolaryngol.

[23] Stachler RJ, Francis DO, Schwartz SR (2018). Clinical practice guideline: hoarseness (dysphonia) (update). Otolaryngol–Head Neck Surg..

[24] Craig P, Dieppe P, Macintyre S (2008). Developing and evaluating complex interventions: the new Medical Research Council guidance. BMJ.

[25] Craig P, Petticrew M (2012). Developing and evaluating complex interventions: reflections on the 2008 MRC guidance. Int J Nurs Stud.

[26] Skivington K, Matthews L, Simpson SA, et al. A new framework for developing and evaluating complex interventions: update of Medical Research Council guidance. Br Med J. 2021;371:n2061. 10.1136/bmj.n2061 PMC8482308 34593508

[27] Branski RC, Verdolini K, Sandulache V (2006). Vocal fold wound healing: a review for clinicians. J Voice.

[28] Branski RC, Rosen CA, Verdolini K (2005). Biochemical markers associated with acute vocal fold wound healing: a rabbit model. J Voice.

[29] Kaneko M, Kishimoto Y, Suzuki R (2017). Protective effect of astaxanthin on vocal fold injury and inflammation due to vocal loading: a clinical trial. J Voice.

[30] Keylock KT, Vieira VJ, Wallig MA (2008). Exercise accelerates cutaneous wound healing and decreases wound inflammation in aged mice. Am J Physiol Regul Integr Comp Physiol.

[31] Cantu R, Steffe JA. (2013).

[32] Bergan CC. (2010). Motor learning principles and voice pedagogy: theory and practice. J Singing.

[33] Wenke RJ, Stabler P, Walton C (2014). Is more intensive better? Client and service provider outcomes for intensive versus standard therapy schedules for functional voice disorders. J Voice.

[34] Wenke R, Coman L, Walton C, Madill C, Theodoros D, Bishop C, Stabler P, Lawrie M, O'Neill J, Gray H, Cardell EA. Effectiveness of Intensive Voice Therapy Versus Weekly Therapy for Muscle Tension Dysphonia: A Noninferiority Randomised Controlled Trial With Nested Focus Group. J Voice. 2021:S0892-1997(21)00064-3. 10.1016/j.jvoice.2021.02.011. Epub ahead of print. PMID: 33741236. 33741236

[35] Michie S. (2014).

[36] Michie S, Richardson M, Johnston M (2013). The behavior change technique taxonomy (v1) of 93 hierarchically clustered techniques: building an international consensus for the reporting of behavior change interventions. Ann Behav Med.

[37] Govender R, Smith CH, Taylor SA (2017). Swallowing interventions for the treatment of dysphagia after head and neck cancer: a systematic review of behavioural strategies used to promote patient adherence to swallowing exercises. BMC Cancer.

[38] White A, Carding P, Booth V, Logan P. Pre- and post-operative voice therapy (PaPOV): Development of an intervention for patients with benign vocal fold lesions. Int J Lang Commun Disord. 2022. 10.1111/1460-6984.12771. Epub ahead of print. PMID: 36047250. PMC10086784 36047250

[39] Hoffmann TC, Glasziou PP, Boutron I (2014). Better reporting of interventions: template for intervention description and replication (TIDieR) checklist and guide. BMJ: Br Med J.

[40] Keeney S, Hasson F, McKenna H (West Sussex, United Kingdom; 2010).

[41] Hasson F, Keeney S, McKenna H. (2000). Research guidelines for the Delphi survey technique. J Adv Nurs.

[42] Boulkedid R, Hendy A, Marine L (2011). Using and reporting the Delphi method for selecting healthcare quality indicators: a systematic review. PLoS One.

[43] White A, Carding P (2022). Pre and post-operative voice therapy: a UK survey of current practice. Bull Off Mag R Coll Speech Lang Ther. Issue.

[44] Van Stan J, Whyte J, Duffy J (2019). Using the rehabilitation treatment specification system to identify targets/ingredients in vocal rehabilitation. Arch Phys Med Rehabil.

[45] Inc Z. Speech/Language Therapist Demographics and Statistics in the US 2022. Available at: https://www.zippia.com/speech-language-therapist-jobs/demographics/. Accessed 24 August 2022.

[46] Van Stan JH, Whyte J, Duffy JR (2021). Voice therapy according to the rehabilitation treatment specification system: expert consensus ingredients and targets. Am J Speech Lang Pathol.

[47] Van Stan JH, Roy N, Awan S (2015). A taxonomy of voice therapy. Am J Speech Lang Pathol.

[48] Van Stan JH, Whyte J, Duffy JR (2021). Rehabilitation treatment specification system: methodology to identify and describe unique targets and ingredients. Arch Phys Med Rehabil.

[49] Nix J. (2017). Best practices: using exercise physiology and motor learning principles in the teaching studio and the practice room. J Singing.

[50] Madill C, McIlwaine A, Russell R (2020). Classifying and identifying motor learning behaviors in voice-therapy clinician-client interactions: a proposed motor learning classification framework. J Voice.

[51] Meerschman I, Claeys S, Bettens K (2019). Massed versus spaced practice in vocology: effect of a short-term intensive voice therapy versus a long-term traditional voice therapy. J Speech Lang Hear Res.

[52] Kaneko M, Shiromoto O, Fujiu-Kurachi M (2017). Optimal duration for voice rest after vocal fold surgery: randomized controlled clinical study. J Voice.

[53] Kiagiadaki D, Remacle M, Lawson G (2015). The effect of voice rest on the outcome of phonosurgery for benign laryngeal lesions: preliminary results of a prospective randomized study. Ann Otol Rhinol Laryngol.

[54] Björck G, Hertegård S, Ekelund J (2022). Voice rest after vocal fold polyp surgery: a Swedish register study of 588 patients. Laryngosc Invest Otolaryngol.

[55] Whitling S, Lyberg-Åhlander V, Rydell R. (2018). Absolute or relative voice rest after phonosurgery: a blind randomized prospective clinical trial. Logoped Phoniatr Vocol.

[56] King RE, Novaleski CK, Rousseau B. (2022). Voice handicap index changes after microflap surgery for benign vocal fold lesions are not associated with recommended absolute voice rest duration. Am J Speech Lang Pathol.

[57] Verdolini Abbott K, Li NYK, Branski RC (2012). Vocal exercise may attenuate acute vocal fold inflammation. J Voice.

[58] Li NYK, Vodovotz Y, Kim KH (2011). Biosimulation of acute phonotrauma: an extended model. Laryngoscope.

[59] Zeitels SM, Hillman RE, Mauri M (2002). Phonomicrosurgery in singers and performing artists: treatment outcomes, management theories, and future directions. Ann Otol Rhinol Laryngol.

[60] Zeitels SM. (2019). The art and craft of phonomicrosurgery in grammy award-winning elite performers. Ann Otol Rhinol Laryngol.

[61] Behrman A. (2006). Facilitating behavioral change in voice therapy: the relevance of motivational interviewing. Am J Speech Lang Pathol.

[62] Hartmann-Boyce J, Johns DJ, Jebb SA (2014). Effect of behavioural techniques and delivery mode on effectiveness of weight management: systematic review, meta-analysis and meta-regression. Obes Rev.

[63] Hartmann-Boyce J, Hong B, Livingstone-Banks J (2019). Additional behavioural support as an adjunct to pharmacotherapy for smoking cessation. Cochrane Database Syst Rev.

[64] Belton I, MacDonald A, Wright G (2019). Improving the practical application of the Delphi method in group-based judgment: a six-step prescription for a well-founded and defensible process. Technol Forecast Social Change.

[65] Okoli C, Pawlowski SD. (2004). The Delphi method as a research tool: an example, design considerations and applications. Inform Manage.

