# Supplementary material for: Pre- and Postoperative Voice Therapy for Benign Vocal Fold Lesions: An International Electronic Delphi Consensus Study
Source: J Voice. 2025 May;39(3):664–75. doi: 10.1016/j.jvoice.2022.12.008 (PMC12063773; doi:10.1016/j.jvoice.2022.12.008)
Supplement: Supplementary file 1 [file mmc1.docx]

**Appendix A: Round 1 results**

| **Statement** | **Mean (SD)** | **Summary of quantitative results** | **Summary of qualitative results** |
| --- | --- | --- | --- |
| **Statement 1) Patients who are undergoing phonosurgery for benign vocal fold lesions should be offered pre- and post-operative voice therapy.**  Explanatory statement: Patients will always have the option to opt out of an intervention but the opportunity to see a specialist voice therapist before and after a surgical procedure should be offered | **4.73**  **(0.688)** | **97.8% Agree or Strongly Agree**   - Strongly Agree 80% - Agree 17.8% - Neither agree nor disagree 0% - Disagree 0% | Participants strongly advocated for SLT involvement both pre and post operatively. There were many comments relating to the role of pre-operative intervention. Participants commented that pre surgical input was essential for sharing information and education regarding voice care, factors contributing to the development of the lesion, establishing goals, patient priorities and expectations, along with addressing muscle tension elements and phonotraumtic behaviours. All of these factors would be followed up in early post-operative voice therapy. |
| **Statement 2) Following phonosurgery, a balance must be achieved between principles of voice rest (wound healing) and remobilisation of the epithelium.**  Explanatory statement: Following phonosurgery, there is a wound of varying depth and length on the vocal fold. The initial stage of wound healing occurs within 72 hours, with further healing processes extending for longer periods. Remobilisation refers to the principle of resuming limited voice use and gentle vocal exercises to re-establish mucosal wave vibration. | **4.80**  **(0.457)** | **97.8% Agree or Strongly Agree**   - Strongly Agree 82.2% - Agree 15.6% - Neither agree nor disagree 2.2% - Disagree 0% - Strongly disagree 0% | Participants overwhelmingly agreed with this principle. Some referred to literature on wound healing and timescales for re-epithelialisation pointing towards a short period of voice rest. Participants cited evidence from acute vocal fold inflammation which favoured early gentle vocal exercise, improved functional outcomes with early vocal fold mobilisation, reduced adherence and increased psychological implications associated with prolonged voice rest. Some participants described how their current practice aligned to this statement e.g. Usually 2 days of complete voice rest then 5 days of relative voice rest with pre-taught gentle vocal exercises. One participant explained that in their institute this 'balance' started from the point of surgery, with no complete voice rest. Specific scenarios, e.g. lesions involving the anterior commisure may impact this balance in some centres. The participant who responded neither agree nor disagree remarked that it was not clear from the statement at which point voice therapy should start but described their practice of 72 hours complete voice rest prior to starting gentle exercises. |
| **Statement 3) Providing opportunities to practice appropriate voice use through regular exercises and functional tasks will contribute to learning new vocal skills.**  Explanatory statement: Motor and sensory representations of a vocal activity are developed through repetition. Learning new vocal skills requires long term retention of those representations and generalisation of the activity to similar tasks and situations | **4.67**  **(0.522)** | **97.8% Agree or Strongly Agree**   - Strongly Agree 68.9% - Agree 28.9% - Neither agree nor disagree 2.2% - Disagree 0% - Strongly disagree 0% | Most participants strongly agreed with this statement, explaining that it was supported by motor learning theory. Some participants felt that the more functional a task the better. There was an acknowledgement that some patients had greater spontaneous carryover of a target voice into functional situations than others. One participant who agreed with the statement clarified that in this population, the rationale was not only to teach a patient how to voice better and reinforce healthy voicing but also to promote healing. Some participants gave examples of how this might be done e.g. relating to the timing - 'little and often', 'before and after high vocal loads' or the kind of feedback given 'using multi level feedback', 'use of negative practice'. |
| **Statement 4) Information should be available to patients in multiple modalities where possible.**  Explanatory statement: Examples of information sharing through multiple modalities include written (paper), written (digital), verbal, watched or read presentation slides, video education clips | **4.49**  **(0.727)** | **91.1% Agree or Strongly Agree**   - Strongly Agree 60% - Agree 31.1% - Neither agree nor disagree 6.7% - Disagree 2.2% - Strongly disagree 0% | Most participants strongly agreed with this statement. Participants explained that patients have different learning styles and different preferences on how to receive information and advice. Having multiple options increased patient understanding and retention especially in scenarios where patients had barriers to accessing information. Some participants described the formats that they used which included a mix of generic and customised information in multiple formats "leaflets, tailored written exercises, demonstration videos, tailored voice recordings and of course, written instructions". One participant explained that regardless of the modality (written/verbal), all information should have a consistent message. One participant disagreed with this statement, stating that they did not like the idea of generic handouts or video education clips and that information should be individually tailored. |
| **Statement 5) A pre-operative checklist should include advice regarding vocal hygiene, voice conservation, reflux management, and communication strategies for use during post-operative voice rest.**  Explanatory Statement: A checklist will offer specific suggestions to improve preparation for surgery. Examples include: 1) Asking the patient to follow pharmacological and lifestyle advice for laryngopharyngeal reflux rigorously to reduce acute inflammation. 2) Suggesting friends communicate with the patient via text and email rather than verbally in the immediate post-operative period. | **4.69**  **(0.514)** | **97.8% Agree or Strongly Agree**   - Strongly Agree 71.1% - Agree 26.7% - Neither agree nor disagree 2.2% - Disagree 0% - Strongly disagree 0% | Most participants strongly agreed with this statement. Additional comments referred to the importance of patients receiving information early to optimise their surgical outcomes, the benefits of written information highlighting key points, and the option of personalising a checklist according to individual circumstances to make the advice as bespoke as possible. One participant agreed with the statement but felt caution was needed to ensure that a 'checklist' was not rushed through and it was used as a basis of discussion. A couple of participants remarked that reflux was at times overdiagnosed but should be included where relevant and that advice regarding throat irritation could instead be used for the majority of patients. The one participant who neither agreed nor disagreed echoed others' views that the example of reflux management should only be used if the patient was symptomatic. |
| **Statement 6) Written post-operative voice use guidance should include graded tasks with examples of voice use at different time points in rehabilitation.** Explanatory Statement: Grading of tasks would consider the length of time spent voicing and the style of task (e.g. confidential voice versus projection). Different time points relates to the time since phonosurgery. This will be considered in relation to the patient's vocal skill, type of surgery and presence of undesirable sensory symptoms (e.g. pain). | **4.11**  **(0.935)** | **75.5% Agree or Strongly Agree**   - Strongly Agree 42.2% - Agree 33.3% - Neither agree nor disagree 17.8% - Disagree 6.7% - Strongly disagree 0% | There was a broader range of responses to this question but the most common response was strongly agree. Those who strongly agreed and agreed felt that specific guidance was helpful, could be customised to the individual and developed alongside surgical colleagues depending on the nature of the surgery and individual vocal requirements. Grading of tasks could help fearful patients and establish expectations for patients returning to work. Specific examples of vocal tasks could help patients to relate situations to their own voice use. Those who disagreed or neither agreed nor disagreed echoed similar views to those in agreement. They stated that collaboration with the phonosurgeon was required and that guidance would need to be individualised. Additionally, they felt that increased focus on undesirable sensory symptoms should be given priority over time since surgery. |
| **Statement 7) Video clips of the patient or clinician performing the voice exercises prescribed should be provided to support home practice.**  Explanatory comment: Video clips could include professionally recorded voice exercises or short recordings made during the voice therapy session to serve as a reminder of the target voice. | **3.89**  **(0.832)** | **68.8% Agree or Strongly Agree**   - Strongly Agree 24.4% - Agree 44.4% - Neither agree nor disagree26.7 % - Disagree 4.4% - Strongly disagree 0% | Many participants discussed the benefits of using videos, including reduced errors and quicker progression between sessions. Feedback from patients was had been positive when videos were introduced. Some felt that video was essential to visualise the whole body, whilst others thought sound files worked adequately. Constraints on sharing of large files restricted this practice for some and some participants favoured individually created videos to generic voice exercise videos. Many participants reflected that patients had individual learning styles and that use of videos was dependent on the patient's need. |
| **Statement 8) A personalised goal setting sheet should supplement generic advice sheets, to optimise compliance by identifying barriers and facilitators relevant to the patient's situation.**  Explanatory comment: The clinician will use the feedback and experiences of the patient between sessions to generate personalised plans. Examples include: 1) Identifying suitable times during the day to drink additional water or 2) developing classroom strategies to reduce shouting. | **4.27**  **(0.863)** | **82.2% Agree or Strongly Agree**   - Strongly Agree 48.9% - Agree 33.3% - Neither agree nor disagree 13.3% - Disagree 4.4% - Strongly disagree 0% | Most participants strongly agreed with this statement. Those who strongly agreed, commented that individualisation of advice and goals was essential. Participants described that this process enabled patients to see how goals could be realistically implemented and felt this kind of problem solving and individualisation of advice was a basic therapeutic practice. Some participants gave examples of barriers and facilitators used with individual patients. A number of participants acknowledged that this could be done in different formats - e.g. annotating advice sheets or emailing additional notes and suggestions after sessions.  Those who neither agreed nor disagreed, felt that whilst this could be helpful, it was "certainly not a necessary tool" as they felt most post-operative patients were highly motivated. One participant felt that as patients have lived experience of their voice problem, they might not need a written reminder of what they want to achieve. Two participants disagreed. One felt it was patient dependent and the other felt it was not the job of the therapist to create a personalised goal setting sheet. |
| **Statement 9) A record sheet to document exercise practice and vocal hygiene adherence will improve accountability, motivation and compliance.**  Examples include: 1) a smartphone App tracking the number of glasses of water consumed in a day or 2) a written sheet with boxes for the patient to tick when vocal exercises have been completed. | **3.73**  **(0.780)** | **57.8% Agree or Strongly Agree**   - Strongly Agree 17.8 % - Agree 40% - Neither agree nor disagree 40% - Disagree 2.2% - Strongly disagree 0% | Some participants felt this was a very useful, even essential tool whilst others felt it could increase patient burden. Some patients would see it as helpful and motivating but others would find it increased anxiety and caused the patient to focus overly on symptoms. In some patients it could lead to disengagement. Some participants discussed different motivation styles and the benefits of placing the responsibility for compliance on the patient by setting collaborative goals. |
| **Statement 10) Developing the discrimination skills to detect and monitor changes in voice quality is an essential component of voice therapy in this population.**  Explanatory Statement: Discrimination skills refers to the patient's ability to perceive differences in their own voice or a clinician model. Changes in voice quality requires that patients understand concepts such as roughness, breathiness and strain. Example: The patient produces a steady hum and comments on whether they can hear any roughness (turbulence) in their voice. | **4.29**  **(0.787)** | **88.8% Agree or Strongly Agree**   - Strongly Agree 44.4% - Agree 44.4% - Neither agree nor disagree 6.7% - Disagree 4.4% - Strongly disagree 0% | Most participants agreed or strongly agreed with this statement and felt improved self-awareness and monitoring of voice quality was helpful but when learning a voice exercise and also when monitoring their voice in functional situations. It enabled independence and the possibility for the patient to 'become their own therapist'. Many felt that auditory self-discrimination was used in conjunction with sensory awareness. Those who neither agreed nor disagreed felt that pre-operatively there was a greater focus on 'feeling' a change but that post-operatively both discrimination skills for voice quality and sensations were important. One participant who disagreed remarked that some patients could not hear a difference and the other commented that if the voice was back to normal after surgery, then they did not need to know in depth about their voice. |
| **Statement 11) Developing the discrimination skills to be able to detect and monitor volume changes in the voice is an essential component of voice therapy in this population.**  Explanatory Statement: Discrimination skills relating to volume refers to the patient's ability to judge how loud or quiet they are. This may be relative to a target, or a comparison volume, in their own or a clinician's model. Example: The clinician produces a voice at different volumes and asks the patient to identify the appropriate volume for their gentle post-operative voice exercise practice. | **4.13**  **(0.786)** | **84.4% Agree or Strongly Agree**   - Strongly Agree 33.3% - Agree 51.1% - Neither agree nor disagree 11.1% - Disagree 4.4% - Strongly disagree 0% | The most common response to this statement was agree. Participants who agreed or strongly agreed with this statement felt that volume discrimination was essential in complying with moderating their own voice in the early post-operative period. Many participants felt that the use of Apps with decibel monitors may be helpful for some patients. A couple of participants who agreed, acknowledged that this was only important for some patients, a theme picked up by those who neither agreed nor disagreed. One participant who disagreed with this statement, acknowledged that developing the discrimination skills to monitor volume was important in the immediate post-operative period to prevent vocal harm, but preferred to focus more on sensation. |
| **Statement 12) Developing the skills to be able to detect and monitor changes in vocal tract resonance is an essential component of voice therapy in this population.**  Explanatory Statement: Discrimination skills relate here to the resonant quality of voice. Example: The patient practices vocalising with different jaw and tongue positions to developing the kinaesthetic awareness to discriminate between 'forward' resonance, involving vibratory sensations on the alveolar ridge and other facial bones versus backed resonant quality where these vibratory sensations will be absent. | **4.24**  **(0.802)** | **86.6% Agree or Strongly Agree**   - Strongly Agree 42.2% - Agree 44.4% - Neither agree nor disagree 8.9% - Disagree 4.4% - Strongly disagree 0% | Most participants strongly agreed or agreed with this statement. Those who agreed or strongly agreed remarked that these skills could aid self-monitoring. Some described using negative practice to help tune into alterations in resonance and used strategies to generate additional feedback such as cupping hands, feeling the buzz in the lips, or flutter int he cheeks with water resistance. One participant who strongly agreed cautioned against overly focusing on details such as tongue and jaw position which for some could make it harder.  Those who neither agreed nor disagreed and those who disagreed felt that this was only sometimes used. One participant preferred to work on volume, and another said they only sometimes used forward focus of tone as a therapeutic target. This participant described the use of other exercises which indirectly impacted resonance through alteration of the vocal tract shape but were not focused on the patient's ability to detect and monitor changes. |
| **Statement 13) A period of absolute voice rest, including avoidance of all laryngeal valving activities should be recommended following phonosurgery.**  Explanatory Statement: Absolute voice rest (sometimes termed 'complete voice rest') means no voicing at all. Laryngeal valving activities include, but are not limited to, all types of phonation, coughing, throat clearing, straining, lifting, and grunting. | **4.13**  **(1.036)** | **77.8% Agree or Strongly Agree**   - Strongly Agree 46.7% - Agree 31.1% - Neither agree nor disagree 13.3% - Disagree 6.7% - Strongly disagree 2.2% | The most common response to this statement was strongly agree, followed by agree. However, there was a full spread of responses with 3 participants disagreeing and 1 strongly disagreeing with this statement. The participant who strongly disagreed felt that use of a very soft voice should be used from the start and one participant who disagreed commented that the duration of absolute voice rest was variable. A theme around variability continued. One participant who neither agreed nor disagreed reported that this was lesion dependent with deeper lesions potentially benefiting from only relative voice rest to stretch the scar.  Those participants who agreed or strongly agreed frequently commented on their local practice. Typically this was to request 24-72 hours of absolute voice rest, with one centre recommending 7 days. Some participants linked this to evidence on wound healing, surgeon preference, the wish to avoid prolonged voice rest and some acknowledged exceptions where they felt absolute voice rest was not required. |
| **Statement 14) A period of relative voice rest should be recommended following phonosurgery.**  Explanatory Statement: Relative voice use involves reducing the frequency of phonation, the duration of voice use, and the intensity/volume of vocalisations. Relative voice use may be used as a follow on from absolute voice rest or as an alternative to absolute voice rest in this statement. | **4.51**  **(0.787)** | **93.3% Agree or Strongly Agree**   - Strongly Agree 62.2% - Agree 31.1% - Neither agree nor disagree 4.4% - Disagree 0% - Strongly disagree 2.2% | Most participants strongly agreed with this statement, with many other agreeing. Those who agreed or strongly agreed reported that they felt comfortable recommending this in principle though had uncertainties about specific timescales. Some participants felt 1-2 weeks relative voice rest was usually recommended. Others talked about grading up activities and planning vocal activities particularly for professional voice users. A large number of participants reported that relative voice rest followed a short period of absolute voice rest. One participant commented that patients should be given a full explanation of what was meant by the term and another commented that the patient should recognise that the voice may not sound normal when they first started to talk.  One participant who selected neither agree nor disagree felt this should be decided by the phonosurgeon and the one participant who strongly disagreed explained "After surgery absolute voice rest is indicated". This is an example where the statement and supporting comment, have been misunderstood or misread by the participant as the statement reads "relative voice use may be used as a follow on from absolute voice rest." |
| **Statement 15) Patients should be encouraged to resume gentle vocalisation within the first week following phonosurgery.**  Explanatory Statement: This relates to the resumption of some level of vocal activities within a week (relative voice rest) where a patient has been following a programme of absolute voice rest. | **4.42**  **(0.723)** | **91.1% Agree or Strongly Agree**   - Strongly Agree 53.3% - Agree 37.8% - Neither agree nor disagree 6.7% - Disagree 2.2% - Strongly disagree 0% | Most participants strongly agreed with this statement, with many other agreeing. Those who strongly agreed or agreed based this on their understanding of the benefits of early mobilisation, results of clinical practice and in discussion with the phonosurgical colleagues. Participants talked about using gentle semi-occluded vocal tract exercises and brief conversational responses in the early post-operative period. One participant agreed but said voicing should only be initiated after ENT review of the vocal folds. Some participants raised questions about the amount of voice use and the balance between use of an exercise v's functional conversations with friends and family. One participant who neither agreed nor disagreed commented that the decision was based on the surgery, the patient's recovery and in liaison with the surgical team. There were no other comments from those who neither agreed nor disagreed, nor from the participant who disagreed. |
| **Statement 16) Giving patients the opportunity to practice pitch glides within the first two weeks following phonosurgery is beneficial.**  Explanatory Comment: Pitch glides involve producing a vocalisation whilst making a smooth transition in pitch. Example: Instruct the patient to alter the pitch of a vocalisation (e.g. lip trill, semi-occluded vowel) in a smooth transition by first raising then lowering the pitch. The patient is told to incrementally increase the pitch glide according to established success criteria. | **4.09**  **(0.848)** | **73.4% Agree or Strongly Agree**   - Strongly Agree 37.8% - Agree 35.6% - Neither agree nor disagree 24.4% - Disagree 2.2% - Strongly disagree 0% | Some participants reported that they felt uncertain about the evidence base for using glides in the early post-operative period. This stopped some from recommending it. Others recommended glides and found these beneficial but were uncertain of the underlying mechanism. Some participants referred to wound healing processes and the potential benefits of low force stretch and contract exercises following vocal fold injury. Participants reiterated the need to use SOVT sounds, smooth transitions (glissando) and close self-monitoring to maintain low effort and low volume when starting these exercises. |
| **Statement 17) Giving patients the opportunity to practice increased levels of muscle activation during vocalisation in exercises and speech tasks post-operatively is beneficial.**  Explanatory Statement: Increased levels of muscle activation includes vocal projection. Example: Teach exercises which focus on safe methods of projection either for infrequent use e.g. calling to the dog, or for sustained use e.g. with a school teacher. | **4.07**  **(0.809)** | **80% Agree or Strongly Agree**   - Strongly Agree 31.1% - Agree 48.9% - Neither agree nor disagree 15.6% - Disagree 4.4% - Strongly disagree 0% | The most common response to this statement was agree, followed by strongly agree. Those who agreed or strongly agreed commented that this should always be offered, following a hierarchical approach, depending on the vocal recovery and making the tasks relevant to the individuals lifestyle requirements. Many acknowledged that confidence in vocal fold healing was a pre-requisite and that timescales would be variable. One participant explained that it was important to include projection work as use of inefficient voice may be a significant contributory factor in lesion development.  One participant reported that time constraints in therapy would sometime limit the amount of time spent on projection. Two participants commented on the benefits of starting safe vocal projection work pre-operatively as "one can never know when a period of shouting may be necessary". Those who neither agreed nor disagreed and those who disagreed, did teach projection techniques but commented that they would not want to do this in the early post operative period, with one participant adding a timescale of 4-6 weeks before starting to train safe projection. |
| **Statement 18) Semi-occluded vocal tract (SOVT) exercises using an anatomical structure or external vehicle are a beneficial component of voice therapy in this population**  Explanatory Statement: SOVT exercises provide an increased dose of resistance. Examples of SOVT exercises using an anatomical structure include lip trills, tongue trills, semi-occluded vocals, voiced fricatives or nasal consonants. Examples of external delivery vehicles include tubing, straw, kazoo, hand over mouth and flow ball devices. | **4.58**  **(0.690)** | **93.4% Agree or Strongly Agree**   - Strongly Agree 66.7% - Agree 26.7% - Neither agree nor disagree 4.4% - Disagree 2.2% - Strongly disagree 0% | The majority of participants strongly agreed with this statement. Those who strongly agreed commented that evidence strongly supported the used of these exercises in healthy and dysphonic patients and that this matched clinical experience in patients with BVFLs. They referred to the reduced collision forces during vibration reported in the literature and clinical experience of observing efficient vocal patterns, release of tension, improved mucosal wave as seen on stroboscopy, and the ability to comfortably glide on a tone. A number of participants commented on the range of SOVTs, which generally was seen as beneficial as the effect of an exercises would be patient dependent and there were many options to trial. However, one participant commented that different levels of back pressure  across different exercises and intensities may impact the amplitude of the vibration and it was important for clinicians to be aware of this when choosing an appropriate exercise/volume level in the very early post-operative recovery. One participant who neither agreed nor disagreed commented that they would not use high levels of resistance in the immediate post-surgical period but would introduce a little resistance once healing was progressing. No other comments were made. |
| **Statement 19) Augmentative feedback tools using external devices which deliver visual or auditory biofeedback are a beneficial component of voice therapy in this population.**  Explanatory Comment: Examples of devices include laryngeal endoscopy, surface electromyography, and microphones delivering an auditory or visual cue when target production is achieved/missed. | **3.73**  **(0.963)** | **55.6% Agree or Strongly Agree**   - Strongly Agree 26.7% - Agree 28.9% - Neither agree nor disagree 35.6% - Disagree 8.9% - Strongly disagree 0% | Participants remarked that decisions regarding use of feedback devices was patient dependent. Additional tools were not always essential, and that access could be difficult. Devices giving visual feedback should not replace therapy to improve a patient's own discriminatory skills.  Laryngeal endoscopy was seen as favourable by several participants, and under used by some. Endoscopy could give the patient improved information, impacting on compliance. One participant commented that endoscopy could help to target rehabilitation techniques effectively, especially in this population where wound healing timeframes could be variable. |
| **Statement 20) Breathing techniques are an essential component of pre and post-operative voice therapy.**  Explanatory comment: Breathing techniques may include all that are theoretically relevant to that patient, including vegetative breathing, oral versus nasal breathing, airflow without voicing, co-ordination of voice and breath. The target of these techniques may be to improve respiratory co-ordination or abdominal movement during breathing. | **4.00**  **(0.977)** | **71.1% Agree or Strongly Agree**   - Strongly Agree37.8 % - Agree 33.3% - Neither agree nor disagree 20% - Disagree 8.9% - Strongly disagree 0% | Most participants felt that intervention targeting breathing was appropriate for some if not all patients. Participants commented that achieving optimal co-ordination of voice and breath could be done without direct focus on breathing exercises and could be encompassed within a holistic therapy technique. The level of intervention was patient dependent following assessment and observation. A couple of participants expressed a cautionary tone, explaining that direct focus on breathing could be counterproductive and in some could increase laryngeal resistance. Others reiterated that any exercise should encourage low resistance, gentle airflow techniques in the early post-operative period and avoid excessive valving or high collision forces. |
| **Statement 21) Voice amplification devices should be advised for every patient where phonotrauma is suspected to be a contributory factor in the development of the benign vocal fold lesion.**  Explanatory Comment: The target is to give the patient the opportunity to practice voicing with decreased loudness. Example: The patient is advised to use an amplifier in certain situations e.g. the classroom or when giving presentations | **3.02**  **(1.076)** | **33.3% Agree or Strongly Agree**   - Strongly Agree 8.9% - Agree 24.4% - Neither agree nor disagree 33.3% - Disagree 26.7% - Strongly disagree 6.7% | Participants felt that there was a small subgroup of patients who this was useful for, including professional voice users and that amplification could play a role in reducing phonotrauma where prolonged high volume voicing was required. Participants felt that even when it was appropriate to use amplification, this should be done in conjunction with projection work, and modification of environmental factors. |
| **Statement 22) The application of pressure through a described form of laryngeal manual therapy is a beneficial component of pre and post-operative voice therapy.**  Explanatory Comment: The application of pressure through a described form of laryngeal manual therapy is a beneficial component of pre and post-operative voice therapy. | **3.24**  **(0.933)** | **42.3% Agree or Strongly Agree**   - Strongly Agree 6.7% - Agree 35.6% - Neither agree nor disagree 35.6% - Disagree 20% - Strongly disagree 2.2% | Some participants felt that there was a role for manual therapy, alongside other voice therapy techniques in patients who had a muscle tension element to their voice problem. Others felt that this was not often required nor a key component with this population. Specific effectiveness studies with this population were lacking and additional clinician training was essential. Post-operatively it could help to establish proprioceptive and auditory baselines of a released voice allowing the patient to experience a target to aim for and participants reported favourable reports from patients. |
| **Statement 23) Clinicians should use multimodality feedback techniques to enhance a patient’s learning and progression in exercises.**  Explanatory Statement: Multimodality implies more than one form of feedback. Examples include clinician modelling, verbal feedback on performance, visual guidance, tactile feedback. | **4.84**  **(0.367)** | **100% Agree or Strongly Agree**   - Strongly Agree 84.4% - Agree 15.6% - Neither agree nor disagree 0% - Disagree 0% - Strongly disagree 0% | All participants strongly agreed or agreed with this statement. participants commented that this was essential and key to effective therapy. One participant explained that choice of feedback style should reflect the learning style of the patient. They felt it was important for clinicians to have a theoretical understanding of how reinforcement, modelling and repetition work differently. |
| **Statement 24) Clinicians should use a range of strategies to deliver information in a way which maximises patient engagement and adherence in therapy.**  Examples include joint goal setting, problem solving, incentivisation, progression through graded tasks, prompts and cues. This may draw on formal models such as COM-B which focus on delivering information to enhance the patient's Capability, Opportunity and Motivation to perform a desired Behaviour (COM-B). | **4.64**  **(0.645)** | **95.6% Agree or Strongly Agree**   - Strongly Agree 71.1% - Agree 24.4% - Neither agree nor disagree 2.2% - Disagree 2.2% - Strongly disagree 0% | Participants overwhelmingly supported this comment, with some commenting that this was essential, the 'norm', or that they fully agreed with the sentiment. One participant described how it was the therapist's responsibility to share knowledge in the most helpful way with the patient, using a range of tools. Some participants gave examples of strategies used in their clinical practice and one participant explained that this statement linked in well with other therapeutic tools such as active listening, cognitive behavioural therapy, solution focused brief therapy and counselling skills.  The one participant who disagreed with this statement, commented that they "use those that work for that client" implying that it may not be a range of strategies |
| **Statement 25) Continuous clinician assessment of the patient’s presentation and performance will inform the pace and direction of hierarchical tasks.**  Explanatory Statement: Continuous clinician assessment includes perceptual evaluation of voice, analysis of musculoskeletal tension and breathing patterns. Example: if the clinician hears increased roughness in the tone as a patient moves from a cognitively simple to complex task, the clinician may ask the patient to repeat a simpler task in order to allow the patient to experience increased accuracy achieving the target voice. Conversely, where a clinician observes confidence using abdominal breathing patterns at rest, they may swiftly progress to a more challenging activity. | **4.80**  **(0.457)** | **97.8% Agree or Strongly Agree**   - Strongly Agree 82.2% - Agree 15.6% - Neither agree nor disagree 2.2% - Disagree 0% - Strongly disagree 0% | Participants overwhelmingly strongly agreed with this statement commenting that this was the essence of therapy and a core therapeutic principle in all areas of voice therapy. Participants commented on the dynamic nature of therapy and the skill of a clinician to move up and down at a moment's notice depending on the individual's presentation. Furthermore a skilled clinician should support the patient to recognise when they may need to step up/down and advise them that this is both normal and helpful from a motor learning perspective. |
| **Statement 26) The intensity of clinician directed feedback to the patient will be reduced as the patient’s self-evaluation accuracy improves.**  Explanatory Statement: The patient initially gets regular cues and guidance from the clinician whilst they form a representation of how the 'target' voice feels and sounds. E.g. good, that sounds clear, much smoother, use a little more breath. Clinician feedback reduces as the accuracy and confidence of the patient improves, shifting to a patient regulated judgement e.g. "how did that feel? What are you noticing in your voice?" | **4.60**  **(0.780)** | **91.1% Agree or Strongly Agree**   - Strongly Agree 73.3% - Agree 17.8% - Neither agree nor disagree 4.4% - Disagree 4.4% - Strongly disagree 0% | Most participants strongly agreed with this statement. Those who strongly agreed or agreed commented that they typically saw a shift in the patient's ability to self-monitor by the end of therapy but that this was encouraged from the outset. Encouraging this self-monitoring of auditory and kinaesthetic awareness helped patients to manage their vocal symptoms more independently. One participant acknowledged rare exceptions where patients have become fixated on the voice problem and wanted more and more clinician-directed feedback as time went on.  One participant who neither agreed nor disagreed with the statement commented that the wording of the statement made them feel that the therapist's constant feedback is the focus of therapy. Two participants disagreed with the statement. One felt that patients could tune into feedback early on in the process and often didn't need to follow therapist guidance first and the other commented that they would continue to provide clinician feedback on a target voice throughout the therapy process. |
| **Statement 27) Clinicians should recommend a consistent and prescribed dose of exercises to each patient.**  Explanatory comment: Prescribed dose means the same instruction is delivered to every patient. Example: Instruct the patient to practice voicing using exercise A for 2 minutes per hour and exercise B for 1 minute per hour, 6 times during waking hours of the day from day 7. | **2.76**  **(1.246)** | **26.7% Agree or Strongly Agree**   - Strongly agree 11.1% - Agree 15.6% - Neither agree nor disagree 28.9% - Disagree 26.7% - Strongly Disagree 17.8% | Participants advocated the need for individualised dosing which should be patient led. Guidance on dosing was considered important, especially in the first few days of resuming voicing. Participants reported that flexibility to negotiate with patients was important. Identifying a balance between motor learning principles and what was achievable was preferred in the absence of firm evidence on optimal dosing for post-operative patients. |
| **Statement 28) Clinicians and patients should agree an individually tailored dose of exercises.**  Explanatory Statement: Individually tailored dose means that variations from intervention developers' treatment protocols, will occur according to factors such as time since surgery, depth of surgical procedure, vocal requirements etc. Example: Recommending an increased dosage of a pitch glide exercises for a professional singer compared to an unskilled vocalist. | **4.71**  **(0.458)** | **95.5% Agree or Strongly Agree**   - Strongly Agree 73.3% - Agree 22.2% - Neither agree nor disagree 2.2% - Disagree 2.2% - Strongly disagree 0% | The majority of participants strongly agreed with this statement. Those who strongly agreed or agreed commented that a one size fits all approach was not appropriate and that individual tailoring accounted for surgical and patient factors, including site and depth of surgery, vocal requirements and psychosocial factors. Although participants strongly agreed, a couple of participants expressed the desire to have better evidence based guidance on dosing according to surgery or lesion type. This was echoed by the patient who neither agreed nor disagreed with this statement.  One participant disagreed with this statement and commented that exercise dosing should be based on what would assist wound healing best. |
| **Statement 29) Dosing recommendations should optimise muscle memory and habit formation.**  Explanatory Statement: Muscle memory and habit formation refers to the process by which regular repetition of an exercise or task becomes habitual or automatic, thereby requiring less conscious thought. | **2.16**  **(0.976)** | **100% Agree or Strongly Agree**   - Strongly Agree 71.1% - Agree 28.9% - Neither agree nor disagree 0% - Disagree 0% - Strongly disagree 0% | All participants agreed or strongly agreed with this statement, and some provided interesting comments to support and add to this discussion. One participant challenged the use of the term 'muscle memory' preferring to use 'motor pattern', but strongly agreed with the statement in general. Some commented that a principle of 'little and often' worked well and reported that patients grasped this concept easily. The idea of combining exercises with daily activities and functional voice tasks was seen as helpful. One participant mentioned the importance of starting this pre-operatively to establish good habits. One participant felt that SLT/SLPs had a tendency to under rather than over prescribe exercises and others referred to the need to balance regular repetition with tissue healing specifically in this population. |
| **Statement 30) The number of voice therapy sessions should be tailored to the patient's vocal and psychological needs, style of learning, and motivation.**  Examples include: 1) A performer may need additional time to understand the impact of anxiety and emotion on the voice. 2) A teacher may require additional practice to develop stamina and projection techniques | **4.67**  **(0.640)** | **100% Agree or Strongly Agree**   - Strongly Agree 75.6% - Agree 24.4% - Neither agree nor disagree 0% - Disagree 0% - Strongly disagree 0% | All participants agreed or strongly agreed with this statement and recognised the importance individual patient requirements and progression rates. Many additional comments related to service factors which influenced the number of sessions offered. These included resource constraints, caseload numbers, waiting times, caps on the numbers of sessions offered and clinician preferences. |
| **Statement 31) A pre and post-operative voice therapy intervention should have a fixed number of sessions to cover the intervention content.**  Example: Offer 3 pre-operative and 3 post-operative voice therapy sessions for every patient. | **4.76**  **(0.435)** | **11.1% Agree or Strongly Agree**   - Strongly Agree 2.2% - Agree 8.9% - Neither agree nor disagree 15.6% - Disagree 48.9% - Strongly disagree 24.4% | Participants felt that the number of sessions required individual tailoring, according to the patient’s needs. Some participants responded that a fixed minimum number of sessions would be helpful, or that there was merit in having suggested numbers of sessions as ‘standard’ treatment. |
