# Supplementary material for: Pre- and Postoperative Voice Therapy for Benign Vocal Fold Lesions: An International Electronic Delphi Consensus Study
Source: J Voice. 2025 May;39(3):664–75. doi: 10.1016/j.jvoice.2022.12.008 (PMC12063773; doi:10.1016/j.jvoice.2022.12.008)
Supplement: Supplementary file 2 [file mmc2.docx]

**Appendix B: Round 2 results**

| **Statement** | **Mean (SD)** | **Summary of quantitative results** | **Summary of qualitative results** |
| --- | --- | --- | --- |
| **Statement 1: Clinicians should discuss what materials would support an individual patient to practice healthy voice production in exercises between sessions e.g. the provision of video or audio files of pre-taught exercises.**  Explanatory Statement: Use of a target to refer to in between sessions can act as a reminder and reduce errors in home learning for some patients. Individual patient preferences will be considered when choosing methods to support home practice. | **4.36**  (0.892) | **88.6% Agree or Strongly Agree**   - Strongly Agree 54.5% - Agree 34.1% - Neither agree nor disagree 6.8% - Disagree 2.3% - Strongly disagree 2.3% | Most participants strongly agreed with this statement. Those who strongly agreed, commented that this revised statement accounted for individuality, patient led care and collaborative discussions to inform decisions about practice between sessions, all of which could improve adherence. The use of electronic devices to help learning was also in line with many individuals’ lives, using electronic devices as part of their day to day living. One participant who agreed felt that this statement went some way to addressing the problem of lack of feedback whilst out of the clinician’s office.  Those who neither agreed nor disagreed felt that the patient did not need to imitate an exercise in exactly the same way, and another participant felt sometimes, written info was sufficient. The one participant who disagreed felt that audio files alone were not sufficient in the vast majority of patients nor exercises, with the only exception being in the Accent method where removing the visual stimuli was helpful in promoting a hypnotic state of listen and repeat. The participant who strongly disagreed did not offer any elaboration. |
| **Statement 2: Conversations between the clinician and patient should discuss which strategies could improve that patient's engagement, motivation and compliance with home exercise practice and voice care advice.**  Explanatory Statement: One strategy (e.g. tick boxes for exercise completion, or an App to track hydration) may increase or decrease compliance depending on the patient, and barriers and facilitators should be explored. | **4.59**  (0.787) | **93.2% Agreed or strongly agreed**   - Strongly Agree 70.5% - Agree 22.7% - Neither agree nor disagree 4.5% - Disagree 0% - Strongly disagree 2.3% | Most participants strongly agreed with this revised statement considering that it captured the essence of a collaborative, patient centred approach and encouraged patients to be acountable, active participants in the therapeutic process. Some participants felt this aligned with approaches that they used (coaching strategies, solution focused therapy). 2 participants neither agreed nor disagreed. One focused on the demotivating nature of the example (record sheets) given) and the other felt that adherence could be promoted sufficiently through education without the use of additional strategies or techniques. The participant who strongly disagreed did not offer any elaboration. |
| **Statement 3: Pitch glides on semi-occluded vocal tract sounds are useful for encouraging flexibility in the healing vocal fold, provided that effort levels and volume are monitored and remain low and glide ranges are initially restricted to the patient's habitual speaking range fundamental frequency.**  Explanatory Statement: Assessment of the patient's voice quality, close monitoring and feedback will direct the choice of sound (voiced fricative, hum, lip trill) and the progression rate. | **4.18**  (0.756) | **79.5% Agree or Strongly Agree**   - Strongly Agree 38.6% - Agree 40.9% - Neither agree nor disagree 20.5% - Disagree 0% - Strongly disagree 0% | A similar number of participants strongly agreed or agreed with this revised statement. Participants felt that the statement was clearer, with rationale, and many preferred the slightly more cautious tone referencing initial restrictions on the range of the pitch glide. Some who agreed with this statement, did clarify that progression based on quality and feel may be most appropriate for professional voice users even in the early post operative period. 9 participants neither agreed nor disagreed. Some wanted less restrictions on the pitch glides (i.e. quicker progression) and one felt careful monitoring was required to avoid hyperfunction. One participant felt concerned about the implications for wound healing in the acute recovery phase. No participants disagreed or strongly disagreed with this revised statement. |
| **Statement 4: Tools which provide auditory or visual biofeedback can be used to enhance therapy, both within clinical sessions and during home practice, and should be selectively used on an individual basis.**  Explanatory Statement: Decisions will be based on availability, a patients' discriminatory ability and success achieving a target phonation pattern. Examples of devices include software programmes which deliver an auditory or visual cue when the target production is achieved/missed. | **4.39**  (0.618) | **97.7% Agree or Strongly Agree**   - Strongly Agree 43.2% - Agree 54.5% - Neither agree nor disagree 0% - Disagree 2.3% - Strongly disagree 0% | All bar one participant strongly agreed or agreed with this revised statement. The few additional comments supported the term ‘selective use’ and reminded us of the need to improve a patient’s own discriminatory skills where required. The one participant who disagreed with this statement, disagreed with the term ‘selective’ and felt that video laryngeal endoscopy as a tool for biofeedback was essential. |
| **Statement 5: Laryngeal endoscopy helps to improve a patient's understanding of their voice, their disorder, and the efficacy of treatment, which can lead to improved engagement in pre and post-operative voice therapy.**  Explanatory Statement: Decisions regarding the use of laryngeal endoscopy will be based on access, training, and patient preference. | **4.34**  (0.805) | 88.6% Agree or Strongly Agree   - Strongly Agree 50% - Agree 38.6% - Neither agree nor disagree 6.8% - Disagree 4.5% - Strongly Disagree 0% | Half of the participants strongly agreed with this statement and had first-hand experience of the positive outcomes reported by patients following laryngeal endoscopy with teaching and explanation. Some made a distinction between use of endoscopy for education and biofeedback- both of which could be covered by this statement, and both could have benefit. Some supported the statement and remarked it was also of use to the clinician. A couple of participants highlighted the benefits of laryngeal endoscopy with the SLT present or being led by an SLT.  One participant who neither agreed nor disagreed felt that laryngoscopy was only helpful if what was seen was discussed with the patient. Two participants disagreed with the statement. One felt that it was not a natural voicing scenario and therefore never used it post-op therapy and the other felt that some patients had poor tolerance of the scope. They also commented that SLT led laryngeal endoscopy was not routinely available in their country. |
| **Statement 6: Assessment and observation of breathing patterns will influence the choice and degree of direct therapy techniques undertaken with a patient, to optimise co-ordination of breath and voice.**  Explanatory Statement: Observations may include identifying breath holding patterns, clavicular breathing and poor co-ordination of voice and breathing. Direct therapy techniques may be targeted singularly at respiration patterns, or more holistically at co-ordination of breath and voice, depending on the patient. | **4.34**  (0.861) | 86.4% Agree or Strongly Agree   - Strongly Agree 52.3% - Agree 34.1% - Neither agree nor disagree 11.4% - Disagree 0% - Strongly disagree 2.3% | Most participants strongly agreed with this revised statement. Those who strongly agreed commented that this was well worded. The more nuanced statement reinforced the idea that exercise selection should be patient specific. One participant who agreed stated that the patient’s ability to make changes to breathing patterns would also influence the choice of technique. One participant who neither agreed nor disagreed , felt that basic breathing techniques should be applied to all patients and contrastingly another felt there was not the evidence to support breath therapy for patients, preferring intervention to focus on the phonatory level. The participant who strongly disagreed did not provide a comment. |
| **Statement 7: Voice amplification devices can be useful post-operatively for individuals who need increased volume output. However, they should be offered alongside direct therapy techniques to improve vocal technique and in conjunction with other advice to reduce phonotrauma.**  Explanatory Statement: Specific examples of individuals who could benefit include professional voice users who are required to sustain high volume for prolonged periods e.g. a Physical Education (PE) teacher or an individual with a relative with a hearing impairment. Direct therapy techniques include projection techniques (e.g. breath support, twang, or belt) introduced at an appropriate time point post operatively, if a patient's vocal load requires this. | **4.18**  (0.896) | **86.4% Agree or Strongly Agree**   - Strongly Agree 40.9% - Agree 45.5% - Neither agree nor disagree 4.5% - Disagree 9.1% - Strongly disagree 0% | The most common participant response to this revised statement was ‘Agree’ followed by ‘strongly agree’.  Those who supported it felt it was a sensible modification to promote improved vocal technique in addition. Comments supported the use of amplification as an additional option in a proportion of patients. One participant who agreed with the statement felt that selective usage may reduce phono-trauma, manage vocal fatigue, and help psychologically in the post-operative phase. Those who disagreed with this statement referred to financial and resource constraints and one participant felt that patients often used poor technique with external devices so advised against them in the post-operative period. |
| **Statement 8: The application of pressure through a described form of laryngeal manual therapy (LMT) can be a useful additional therapy tool for patients who have intrinsic or extrinsic laryngeal tension contributing to or arising from their benign vocal fold lesion, but it is not currently considered a 'key' component of pre and post-operative voice therapy.**  Explanatory Statement: Laryngeal manual therapy encompasses the application of pressure using a finger/thumb combination/palm or external device such as a vibrator. Pressure is applied using kneading, stroking, static, pulling or oscillation movements to specified laryngeal, neck, or shoulder muscles, during rest or voicing. | **4.09**  (0.772) | **85% Agree or Strongly Agree**   - Strongly Agree 29.5% - Agree 54.5% - Neither agree nor disagree 11.4% - Disagree 4.5% - Strongly disagree 0% | The majority of participants agreed with this revised statement. They acknowledged its use in patients with concomitant MTD. However constraints such as clinician training, patient selection, and the ability to deliver face to face interventions during COVID-19 pandemic, limited it to an additional therapy tool rather than a key component. Some participants who neither agreed nor disagreed, and those who disagreed felt that it was in fact a ‘key’ component for some patients. One participant felt that although the evidence base for LMT in BVFLs was lacking, evidence could be appropriately generalised from the MTD population. Two participants remarked that they would be cautious about using LMT in the post-operative phase but supported its use pre-operatively. |
| **Statement 9: When patients resume vocal activities post-operatively, clinicians should initially recommend individualised but directed practice regimes with dose guidance.**  Explanatory Statement: Timing of the resumption of vocal activities may vary locally according to surgeon preference but specific dosing guidance would remain in place up to two weeks post-operatively during the initial phases of wound healing. | **4.23**  (0.743) | **86.3% Agree or Strongly Agree**   - Strongly Agree 38.6% - Agree 47.7% - Neither agree nor disagree 11.4% - Disagree 2.3% - Strongly disagree 0% | The most common response to this revised statement was ‘agree’ shortly followed by ‘strongly agree’. Those who supported this statement mostly felt that this wording was appropriate given the current uncertainty around dosing. Many felt that short term guidance on dosing in the early post-operative period would encourage habit formation but prevent overuse and that focusing on patients monitoring their own vocal and throat symptoms was important as these would be variable. One participant who agreed with the statement, felt that the term ‘individualised therapy’ was a poor response to the issue and that there was a need to practice in a programmed fashion to get the data to individualise the program to the patient. Those who neither agreed nor disagreed did so for different reasons. One participant’s institution had set doses for techniques and followed these protocols. One felt dosing should be discussed with the phonosurgeon. One felt that guidance would depend on the site of the lesion, and another felt that it was difficult to tailor it to individual patients when the global evidence was unclear, however, felt the premise of gradually increasing consistency in the first two weeks was sensible. |
| **Statement 10: Patients should be encouraged to develop and manage an individual exercise regime which balances principles of motor learning with individual patient circumstances and vocal symptoms.**  Explanatory Statement: Broad principles of motor learning include short, frequent, and varied tasks. Patient circumstances relate to the length of specified absolute and/or relative voice rest, patient goals and vocal load. Vocal symptoms mean being aware of and responding to soreness, strain, fatigue, and /or deterioration in quality. | **4.55**  (0.730) | **90.9% Agree or Strongly Agree**   - Strongly Agree 65.9% - Agree 25% - Neither agree nor disagree 6.8% - Disagree 2.3% - Strongly disagree 0% | Most participants strongly agreed with this revised statement. Participants liked the focus on patient autonomy and empowerment whilst recognising that this often followed a period where clear clinician involvement and guidance was required (see previous statement). Those who neither agreed nor disagreed with the statement did not feel that the wording of the statement was clear. The participant who disagreed did not provide any comment. |
| **Statement 11: A minimum number of 1 pre and 1 post-operative voice therapy sessions could be recommended as a guide for anyone undergoing phonosurgery with the option to increase this according to patient and surgical factors.**  Explanatory Statement: Patient factors may include vocal requirements, preferred learning styles, vocal skill, and level of psychosocial support. Surgical factors will include depth and complexity of surgery. | **4.11**  (1.104) | **81.9% Agree or Strongly Agree**   - Strongly Agree 45.5% - Agree 36.4% - Neither agree nor disagree 6.8% - Disagree 6.8% - Strongly disagree 4.5% | The majority of participants strongly agreed and agreed with this statement, however there was representation across the range of answers. Those who agreed felt that a minimum standard could set an expectation that patients must be referred pre-operatively, which was often lacking and meant patients were undergoing surgery without proper information and education. Participants felt that a minimum standard promoted the involvement and value of voice therapy for these patients. Many participants who strongly agreed or agreed with this statement acknowledged that the number of sessions would increase for many patients and recognised the importance of flexibility. Those who disagreed or strongly disagreed and provided comments felt that the statement should ask for a minimum standard of 1 pre and 3 post-operative sessions or an average of 3 or 4 post operative sessions with the option to increase or decrease. |
