# Supplementary material for: Pre- and Postoperative Voice Therapy for Benign Vocal Fold Lesions: An International Electronic Delphi Consensus Study
Source: J Voice. 2025 May;39(3):664–75. doi: 10.1016/j.jvoice.2022.12.008 (PMC12063773; doi:10.1016/j.jvoice.2022.12.008)
Supplement: Supplementary file 3 [file mmc3.docx]

**Appendix C: Round 3**

*Participants were asked to re-rate statements in round 3. It was acknowledged that they had already shared comments in previous rounds and therefore participants were told that further comments were welcomed but not essential if they felt that they had explained their choices in earlier rounds.*

| **Statement** | **Mean**  **(SD)** | **Summary of quantitative results** | **Summary of qualitative results** |
| --- | --- | --- | --- |
| **Statement 1. Patients who are undergoing phonosurgery for benign vocal fold lesions should be offered pre and post-operative voice therapy.**  Explanatory Statement: Patients will always have the option to opt out of an intervention but the opportunity to see a specialist voice therapist before and after a surgical procedure should be offered | **4.81**  **(0.671)** | **97.6% Agree or Strongly Agree**  Strongly agree 88.1%  Agree 9.5%  Neither Agree nor disagree 0%  Disagree 0%  Strongly disagree 2.4% | Participant comments stated that this was best practice and that all centres should work towards this. One participant discussed the varied ways in which pre-operative input could be very important. Another reported that communication breakdowns and lack of formal pathways sometimes prevented this from happening. One person strongly disagreed with this statement but gave no further explanatory comment. |
| **Statement 2. Following phonosurgery, a balance must be achieved between principles of voice rest (wound healing) and remobilisation of the epithelium.**  Explanatory Statement: Following phonosurgery, there is a wound of varying depth and length on the vocal fold. The initial stage of wound healing occurs within 72 hours, with further healing processes extending for longer periods. Remobilisation refers to the principle of resuming limited voice use and gentle vocal exercises to re-establish mucosal wave vibration | **4.67**  **(0.902)** | **92.5% Agree or Strongly Agree**  Strongly agree 81%  Agree 14.3%  Neither Agree nor disagree 0%  Disagree 0%  Strongly disagree 4.8% | Most participants strongly agreed with this statement. However, comments related to the need for further research to understand the optimal time in different lesion types. One participant commented on the importance of professionals involved in a patient's care giving consistent information. Two participants strongly disagreed with this statement but gave no further comment. |
| **Statement 3. Providing opportunities to practice appropriate voice use through regular exercises and functional tasks will contribute to learning new vocal skills.**  Explanatory Statement: Motor and sensory representations of a vocal activity are developed through repetition. Learning new vocal skills requires long term retention of those representations and generalisation of the activity to similar tasks and situations | **4.74**  **(0.701)** | **97.6% Agree or Strongly Agree**  Strongly agree 88.1%  Agree 9.5%  Neither Agree nor disagree 0%  Disagree 0%  Strongly disagree 2.4% | Participants strongly agreed with this statement. Comments included the importance of using a 'little and often' principle and emphasised the need to include functional tasks relevant to the individual patient, in order to improve carryover into everyday voice use. One participant rated this statement as strongly disagree but did not offer a supporting comment. |
| **Statement 4. Information should be available to patients in multiple modalities where possible.**  Explanatory Statement: Examples of information sharing through multiple modalities include written (paper), written (digital), verbal, watched or read presentation slides, video education clips | **4.69**  **(0.715)** | **97.6% Agree or Strongly Agree**  Strongly agree 76.2%  Agree 21.4%  Neither Agree nor disagree 0%  Disagree 0%  Strongly disagree 2.4% | Participants commented that generic handouts could be seen as a point of reference only, to be built upon and commented that different mediums suited some patients better than others. One participant selected 'strongly disagree' but failed to offer a supporting comment. |
| **Statement 5. A pre-operative checklist should include advice regarding voice care, voice conservation, reflux management, and communication strategies for use during post-operative voice rest.**  A checklist will offer specific suggestions to improve preparation for surgery. Examples include: 1) Asking the patient to follow pharmacological and lifestyle advice for laryngopharyngeal reflux rigorously to reduce acute inflammation. 2) Suggesting friends communicate with the patient via text and email rather than verbally in the immediate post-operative period | **4.50**  **(0.506)** | **100% Agree or Strongly Agree**  Strongly agree 50%  Agree 50%  Neither Agree nor disagree 0%  Disagree 0%  Strongly disagree 0% | All participants agreed with this statement. One highlighted the importance of this being used as a basis for individual patient discussion, rather than a standalone handout. A few participants made the point that information pertaining to reflux management should be given if relevant to that patient. |
| **Statement 6. Written post-operative voice use guidance should include graded tasks with examples of voice use at different time points in rehabilitation.**  Explanatory Statement: Grading of tasks would consider the length of time spent voicing and the style of task (e.g. confidential voice versus projection). Different time points relates to the time since phonosurgery. This will be considered in relation to the patient's vocal skill, type of surgery and presence of undesirable sensory symptoms (e.g. pain). | **4.21**  **(0.645)** | **92.6% Agree or Strongly Agree**  Strongly agree 31%  Agree 61.9%  Neither Agree nor disagree 4.8%  Disagree 2.4%  Strongly disagree 0% | Most participants agreed with this statement, with the second largest amount strongly agreeing. Many comments of participants who agreed, related to the need to use graded tasks as a starting point for further discussion. A couple of participants highlighted that patients should be guided to progress through these tasks according to their vocal and sensory symptoms rather than fixed timepoints. Those who neither disagreed nor agreed, felt that it was hard to have timepoints when there was so much individual variation. |
| **Statement 7. Clinicians should discuss what materials would support an individual patient to practice healthy voice production in exercises between sessions e.g. the provision of video or audio files of pre-taught exercises**  Explanatory Statement: Use of a target to refer to in between sessions can act as a reminder and reduce errors in home learning for some patients. Individual patient preferences will be considered when choosing methods to support home practice. | **4.60**  **(0.734)** | **97.6% Agree or Strongly Agree**  Strongly agree 66.7%  Agree 31%  Neither Agree nor disagree 0%  Disagree 0%  Strongly disagree 2.4% | Participants commented that videos could give additional support and embed an internal locus of control. One participant commented on local challenges with information technology. One participant selected strongly disagree but did not comment further. |
| **Statement 8. A personalised goal setting sheet should supplement generic advice sheets, to optimise compliance by identifying barriers and facilitators relevant to the patient's situation.**  Explanatory Statement: The clinician will use the feedback and experiences of the patient between sessions to generate personalised plans. Examples include: 1) Identifying suitable times during the day to drink additional water or 2) developing classroom strategies to reduce shouting | **4.14**  **(0.843)** | **81% Agree or Strongly Agree**  Strongly agree 38.1%  Agree 42.9%  Neither Agree nor disagree 14.3%  Disagree 4.8%  Strongly disagree 0% | Participants who agreed with this statement felt that individualisation of goals and tasks was important. A couple of participants who agreed felt that it was not always necessary use a sheet, and that this could at times be a verbal discussion. This was also the view of one participant who neither agreed nor disagreed with the statement. One participant who disagreed, referred to the wording in the phrase, considering that personalised notes or instructions was preferable to 'goal setting' |
| **Statement 9: Conversations between the clinician and patient should discuss which strategies could improve that patient's engagement, motivation and compliance with home exercise practice and voice care advice.**  Explanatory Statement: One strategy (e.g. tick boxes for exercise completion, or an App to track hydration) may increase or decrease compliance depending on the patient, and barriers and facilitators should be explored. | **4.62**  **(0.731)** | **97.6% Agree or Strongly Agree**  Strongly agree 69%  Agree 28.6%  Neither Agree nor disagree 0%  Disagree 0%  Strongly disagree 2.4% | Only 3 respondents gave supporting comments, all in favour of this statement. They commented that conversations of this nature encouraged internal locus of control, accountability, could identify motivating strategies and improved patient centred care. |
| **Statement 10. Developing the discrimination skills to detect and monitor changes in voice quality is an essential component of voice therapy in this population.**  Explanatory Statement: Discrimination skills refers to the patient's ability to perceive differences in their own voice or a clinician model. Changes in voice quality requires that patients understand concepts such as roughness, breathiness and strain. Example: The patient produces a steady hum and comments on whether they can hear any roughness (turbulence) in their voice. | **4.43**  **(0.630)** | **92.9% Agree or Strongly Agree**  Strongly agree 50%  Agree 42.9%  Neither Agree nor disagree 7.1%  Disagree 0%  Strongly disagree 0% | Participants who strongly agreed with this statement recognised that some patients found this challenging and that in order for them to make behavioural changes, it was essential that a patient could discriminate between a desirable and undesirable target. Those who agreed added comments that patient's skills were variable and thus more/less time could be required on this. Discrimination around both auditory and sensory symptoms was also acknowledged. |
| **Statement 11. Developing the discrimination skills to be able to detect and monitor volume changes in the voice is an essential component of voice therapy in this population.**  Explanatory Statement: Discrimination skills relating to volume refers to the patient's ability to judge how loud or quiet they are. This may be relative to a target, or a comparison volume, in their own or a clinician's model. Example: The clinician produces a voice at different volumes and asks the patient to identify the appropriate volume for their gentle post-operative voice exercise practice. | **3.95**  **(0.661)** | **88.1% Agree or Strongly Agree**  Strongly agree 11.9%  Agree 76.2%  Neither Agree nor disagree 9.5%  Disagree 0%  Strongly disagree 2.4% | Most participants 'agreed' with this statement, expanding upon the idea that volume was particularly important post-operatively. Others commented that this was most important in patients who were habitually loud, and this was a contributory factor in their lesion development. One person strongly disagreed but failed to give further comment. |
| **Statement 12. Developing the skills to be able to detect and monitor changes in vocal tract resonance is an essential component of voice therapy in this population.**  Explanatory Statement: Discrimination skills relate here to the resonant quality of voice. Example: The patient practices vocalising with different jaw and tongue positions to developing the kinaesthetic awareness to discriminate between 'forward' resonance, involving vibratory sensations on the alveolar ridge and other facial bones versus backed resonant quality where these vibratory sensations will be absent. | **4.12**  **(0.832)** | **88.1% Agree or Strongly Agree**  Strongly agree 31%  Agree 57.1%  Neither Agree nor disagree 7.1%  Disagree 2.4%  Strongly disagree 2.4% | Those who strongly agreed with this statement felt that this would improve a patient's ability to practice a target voice accurately. Others who agreed, felt that this was useful for self-monitoring and that this would be a greater or lesser component of the intervention depending on the patient's specific presentation. Three participants neither agreed nor disagreed with one commenting that this would be patient dependant and linked with other discrimination work. The participant who disagreed felt that voice quality was more important that controlling resonance. |
| **Statement 13) A period of absolute voice rest, including avoidance of all laryngeal valving activities should be recommended following phonosurgery.**  Explanatory Statement: Absolute voice rest (sometimes termed 'complete voice rest') means no voicing at all. Laryngeal valving activities include, but are not limited to, all types of phonation, coughing, throat clearing, straining, lifting, and grunting. | **4.00**  **(0.988)** | **81% Agree or Strongly Agree**  Strongly agree 31%  Agree 50%  Neither Agree nor disagree 11.9%  Disagree 2.4%  Strongly disagree 4.8% | This statement generated the full range of responses, although 81% agreed or strongly agreed. Comments reflected the lack of firm guidance in the literature, the importance of discussions with the operating surgeon and the variability in practice which was dictated by lesion location. Those who neither agreed nor disagreed commented that this depended on the lesion type. One participant also pointed to literature showing poor compliance with absolute voice rest and improved long term functional voice outcomes in those directed to have relative voice rest compared to absolute voice rest. |
| **Statement 14) A period of relative voice rest should be recommended following phonosurgery.**  Explanatory Statement: Relative voice use involves reducing the frequency of phonation, the duration of voice use, and the intensity/volume of vocalisations. Relative voice use may be used as a follow on from absolute voice rest or as an alternative to absolute voice rest in this statement. | **4.50**  **(0.944)** | **92.9% Agree or Strongly Agree**  Strongly agree 66.7%  Agree 26.2%  Neither Agree nor disagree 2.4%  Disagree 0%  Strongly disagree 4.8% | The majority of participants strongly agreed with this statement. Comments suggested offering clear definitions of what RVR meant with examples to patients. Those who agreed commented on the lack of guidance around how long this should be. One participant, may have misunderstood the wording, stating that after surgery absolute voice rest was indicated. |
| **Statement 15) Patients should be encouraged to resume gentle vocalisation within the first week following phonosurgery.**  Explanatory Statement: This relates to the resumption of some level of vocal activities within a week (relative voice rest) where a patient has been following a programme of absolute voice rest. | **4.71**  **(0.508)** | **97.6% Agree or Strongly Agree**  Strongly agree 73.8%  Agree 23.8%  Neither Agree nor disagree 2.4%  Disagree 0%  Strongly disagree 0% | Most participants strongly agreed with this statement. Additional comments clarified the reason for reintroducing the voice - to aid mobilisation, prevent scar, disuse atrophy and encourage new efficient phonation patterns early on. Some commented on joint discussions with the surgeon and SLT. The one participant who neither agreed nor disagreed, felt that reintroduction of voice was always a case by case decision. |
| **Statement 16: Pitch glides on semi-occluded vocal tract sounds are useful for encouraging flexibility in the healing vocal fold, provided that effort levels and volume are monitored and remain low and glide ranges are initially restricted to the patient's habitual speaking range fundamental frequency.**  Explanatory Statement: Assessment of the patient's voice quality, close monitoring and feedback will direct the choice of sound (voiced fricative, hum, lip trill) and the progression rate. | **4.57**  **(0.630)** | **92.9% Agree or Strongly Agree**  Strongly agree 64.3%  Agree 28.6%  Neither Agree nor disagree 7.1%  Disagree 0%  Strongly disagree 0% | Participants who strongly agreed reported that this was fundamental to post operative therapy and that these were used routinely. Others cautioned that monitoring of hyperfunction and determining progression criteria was important. No other comments were made. |
| **Statement 17) Giving patients the opportunity to practice increased levels of muscle activation during vocalisation in exercises and speech tasks post-operatively is beneficial.**  Explanatory Statement: Increased levels of muscle activation includes vocal projection. Example: Teach exercises which focus on safe methods of projection either for infrequent use e.g. calling to the dog, or for sustained use e.g. with a school teacher. | **4.19**  **(0.552)** | **92.9% Agree or Strongly Agree**  Strongly agree 26.2%  Agree 66.7%  Neither Agree nor disagree 7.1%  Disagree 0%  Strongly disagree 0% | Participants mostly agreed with this statement. A couple of participants highlighted the importance of using a hierarchical approach to building volume and one reiterated the importance in this population of considering wound healing stage. One participant questioned whether some SLTs lacked confidence in teaching projection work. One participant who neither agreed nor disagreed, reported that they did not always work on projection directly. |
| **Statement 18) Semi-occluded vocal tract (SOVT) exercises using an anatomical structure or external vehicle are a beneficial component of voice therapy in this population**  Explanatory Statement: SOVT exercises provide an increased dose of resistance. Examples of SOVT exercises using an anatomical structure include lip trills, tongue trills, semi-occluded vocals, voiced fricatives or nasal consonants. Examples of external delivery vehicles include tubing, straw, kazoo, hand over mouth and flow ball devices. | **4.64**  **(0.533)** | **97.6% Agree or Strongly Agree**  Strongly agree 66.7%  Agree 31%  Neither Agree nor disagree 2.4%  Disagree 0%  Strongly disagree 0% | Participants mostly strongly agreed. Only two participants gave further comments, one saying this was fundamental and the other stating that it was important to ensure patients had good technique. |
| **Statement 19: Tools which provide auditory or visual biofeedback can be used to enhance therapy, both within clinical sessions and during home practice, and should be selectively used on an individual basis.**  Explanatory Statement: Decisions will be based on availability, a patients' discriminatory ability and success achieving a target phonation pattern. Examples of devices include software programmes which deliver an auditory or visual cue when the target production is achieved/missed. | **4.19**  **(0.740)** | **92.9% Agree or Strongly Agree**  Strongly agree 31%  Agree 61.9%  Neither Agree nor disagree 4.8%  Disagree 0%  Strongly disagree 2.4% | Most participants agreed with this statement. Only 3 additional comments were made. One, who neither agreed nor disagreed stated they had not used these before, one who agreed, felt that selected use of additional tools was appropriate and one who strongly agreed felt biofeedback improved self-monitoring. |
| **Statement 20: Laryngeal endoscopy helps to improve a patient's understanding of their voice, their disorder, and the efficacy of treatment, which can lead to improved engagement in pre and post-operative voice therapy.**  Explanatory Statement: Decisions regarding the use of laryngeal endoscopy will be based on access, training, and patient preference. | **4.55**  **(0.550)** | **97.6% Agree or Strongly Agree**  Strongly agree 57.1%  Agree 40.5%  Neither Agree nor disagree 2.4%  Disagree 0%  Strongly disagree 0% | This statement had very high levels of agreement. Participants commented that patients often enjoyed seeing their images, that this improved understanding, in turn enhancing compliance and confidence. A number of participants discussed the importance of ensuring there was enough time to discuss images with patients to get the maximum benefit. |
| **Statement 21: Assessment and observation of breathing patterns will influence the choice and degree of direct therapy techniques undertaken with a patient, to optimise co-ordination of breath and voice.**  Explanatory Statement: Observations may include identifying breath holding patterns, clavicular breathing and poor co-ordination of voice and breathing. Direct therapy techniques may be targeted singularly at respiration patterns, or more holistically at co-ordination of breath and voice, depending on the patient. | **4.52**  **(0.634)** | **92.9% Agree or Strongly Agree**  Strongly agree 59.5%  Agree 33.3%  Neither Agree nor disagree 7.1%  Disagree 0%  Strongly disagree 0% | Most participants strongly agreed with this statement. Comments picked up on the individualised assessment which would target specifics of the intervention and the importance of balancing and co-ordinating mechanisms. |
| **Statement 22: Voice amplification devices can be useful post-operatively for individuals who need increased volume output. However, they should be offered alongside direct therapy techniques to improve vocal technique and in conjunction with other advice to reduce phonotrauma.**  Explanatory Statement: Specific examples of individuals who could benefit include professional voice users who are required to sustain high volume for prolonged periods e.g. a Physical Education (PE) teacher or an individual with a relative with a hearing impairment. Direct therapy techniques include projection techniques (e.g. breath support, twang, or belt) introduced at an appropriate time point post operatively, if a patient's vocal load requires this. | **4.31**  **(0.604)** | **92.9 Agree or Strongly Agree**  Strongly agree 38.1%  Agree 54.8%  Neither Agree nor disagree 7.1%  Disagree 0%  Strongly disagree 0% | Participants who agreed with this statement reiterated the benefit of amplification devices for selected individuals. Some gave specific examples of groups who may benefit e.g. public speaking, performers, teachers and to aid return to work. |
| **Statement 23: The application of pressure through a described form of laryngeal manual therapy (LMT) can be a useful additional therapy tool for patients who have intrinsic or extrinsic laryngeal tension contributing to or arising from their benign vocal fold lesion, but it is not currently considered a 'key' component of pre and post-operative voice therapy.**  Explanatory Statement: Laryngeal manual therapy encompasses the application of pressure using a finger/thumb combination/palm or external device such as a vibrator. Pressure is applied using kneading, stroking, static, pulling or oscillation movements to specified laryngeal, neck, or shoulder muscles, during rest or voicing. | **4.07**  **(0.640)** | **88.1% Agree or Strongly Agree**  Strongly agree 21.4%  Agree 66.7%  Neither Agree nor disagree 9.5%  Disagree 2.4%  Strongly disagree 0% | Participants who agreed or strongly agreed with this comment, reiterated the selective nature on which they would use laryngeal manual therapy, based on clinical need and practical purposes. The participant who disagreed with this comment, believed that manual therapy should be a 'key' component. |
| **Statement 24) Clinicians should use multimodality feedback techniques to enhance a patient’s learning and progression in exercises.**  Explanatory Statement: Multimodality implies more than one form of feedback. Examples include clinician modelling, verbal feedback on performance, visual guidance, tactile feedback. | **4.93**  **(0.261)** | **100% Agree or Strongly Agree**  Strongly agree 92.9%  Agree 7.1%  Neither Agree nor disagree 0%  Disagree 0%  Strongly disagree 0% | All participants agreed or strongly agreed, with only two participants providing further elaboration to reinforce the benefits of understanding a patient's preferred learning style. |
| **Statement 25) Clinicians should use a range of strategies to deliver information in a way which maximises patient engagement and adherence in therapy.**  Examples include joint goal setting, problem solving, incentivisation, progression through graded tasks, prompts and cues. This may draw on formal models such as COM-B which focus on delivering information to enhance the patient's Capability, Opportunity and Motivation to perform a desired Behaviour (COM-B). | **4.83**  **(0.660)** | **97.6% Agree or Strongly Agree**  Strongly agree 90.5%  Agree 7.1%  Neither Agree nor disagree 0%  Disagree 0%  Strongly disagree 2.4% | Most participants strongly agreed with this statement. There were only 3 additional comments which reinforced the importance of a patient centred, collaborative approach to therapy. One strongly disagree selection had no further comment to explain their feelings. |
| **Statement 26) Continuous clinician assessment of the patient’s presentation and performance will inform the pace and direction of hierarchical tasks.**  Explanatory Statement: Continuous clinician assessment includes perceptual evaluation of voice, analysis of musculoskeletal tension and breathing patterns. Example: if the clinician hears increased roughness in the tone as a patient moves from a cognitively simple to complex task, the clinician may ask the patient to repeat a simpler task in order to allow the patient to experience increased accuracy achieving the target voice. Conversely, where a clinician observes confidence using abdominal breathing patterns at rest, they may swiftly progress to a more challenging activity. | **4.86**  **(0.647** | **97.6% Agree or Strongly Agree**  Strongly agree 92.9%  Agree 4.8%  Neither Agree nor disagree 0%  Disagree 0%  Strongly disagree 2.4% | Most participants strongly agreed with this statement with only two additional comments. The importance of a hierarchical approach was noted as was the broader application of this comment to other voice therapy populations. One participant who selected 'strongly disagree' gave no further comment to explain their feelings. |
| **Statement 27) The intensity of clinician directed feedback to the patient will be reduced as the patient’s self-evaluation accuracy improves.**  Explanatory Statement: The patient initially gets regular cues and guidance from the clinician whilst they form a representation of how the 'target' voice feels and sounds. E.g. good, that sounds clear, much smoother, use a little more breath. Clinician feedback reduces as the accuracy and confidence of the patient improves, shifting to a patient regulated judgement e.g. "how did that feel? What are you noticing in your voice?" | **4.64**  **(0.759)** | **95.2% Agree or Strongly Agree**  Strongly agree 73.8%  Agree 21.4%  Neither Agree nor disagree 2.4%  Disagree 0%  Strongly disagree 2.4% | Most participants strongly agreed with this statement. Two additional comments reinforced the aim of patients becoming involved in self-monitoring and reflection as therapy progresses. One participant found the wording confusing and therefore rated this as neither agree nor disagree. The participant who strongly disagree, did not comment further. |
| **Statement 28) When patients resume vocal activities post-operatively, clinicians should initially recommend individualised but directed practice regimes with dose guidance.**  Explanatory Statement: Timing of the resumption of vocal activities may vary locally according to surgeon preference but specific dosing guidance would remain in place up to two weeks post-operatively during the initial phases of wound healing. | **4.14**  **(0.472)** | **95.2% Agree or Strongly Agree**  Strongly agree 19%  Agree 76.2%  Neither Agree nor disagree 4.8%  Disagree 0%  Strongly disagree 0% | Participant comments reinforced the idea of the statement and the potential benefit of giving patients specific dosage guidance, but many commented on the lack of evidence to inform this guidance. |
| **Statement 29) Clinicians and patients should agree an individually tailored dose of exercises.**  Explanatory Statement: Individually tailored dose means that variations from intervention developers' treatment protocols, will occur according to factors such as time since surgery, depth of surgical procedure, vocal requirements etc. Example: Recommending an increased dosage of a pitch glide exercises for a professional singer compared to an unskilled vocalist. | **4.64**  **(0.727)** | **95.2% Agree or Strongly Agree**  Strongly agree 73.8%  Agree 21.4%  Neither Agree nor disagree 0%  Disagree 4.8%  Strongly disagree 0% | This statement was met with strong agreement but those who commented, did express a desire for greater evidence to inform dosing guidance. Two participants disagreed. One felt that patients may not have the knowledge to input into this discussion. The other felt that until we had better evidence, we should use standardised regimes and collect data on the outcomes. |
| **Statement 30) Dosing recommendations should optimise muscle memory and habit formation.**  Explanatory Statement: Muscle memory and habit formation refers to the process by which regular repetition of an exercise or task becomes habitual or automatic, thereby requiring less conscious thought. | **4.81**  **(0.397)** | **100% Agree or Strongly Agree**  Strongly agree 81%  Agree 19%  Neither Agree nor disagree 0%  Disagree 0%  Strongly disagree 0% | All participants strongly agreed or agreed. A couple of participants reference a 'little and often' approach which was easy for patients to understand and linked regular practice to principles of neuroplasticity. One participant again, referenced the need for greater evidence to support dosing recommendations. |
| **Statement 31) Patients should be encouraged to develop and manage an individual exercise regime which balances principles of motor learning with individual patient circumstances and vocal symptoms.**  Explanatory Statement: Broad principles of motor learning include short, frequent, and varied tasks. Patient circumstances relate to the length of specified absolute and/or relative voice rest, patient goals and vocal load. Vocal symptoms mean being aware of and responding to soreness, strain, fatigue, and /or deterioration in quality. | **4.60**  **(0.767)** | **90.5% Agree or Strongly Agree**  Strongly agree 69%  Agree 21.4%  Neither Agree nor disagree 2.4%  Disagree 4.8%  Strongly disagree 2.4% | An error on this page initially meant that the first 10 participants who completed the questionnaire saw a different statement. Participants all commented on this and therefore their comments related to another statement. |
| **Statement 32) The number of voice therapy sessions should be tailored to the patient's vocal and psychological needs, style of learning, and motivation.**  Examples include: 1) A performer may need additional time to understand the impact of anxiety and emotion on the voice. 2) A teacher may require additional practice to develop stamina and projection techniques | **4.83**  **(0.660)** | **97.6% Agree or Strongly Agree**  Strongly agree 90.5%  Agree 7.1%  Neither Agree nor disagree 0%  Disagree 0%  Strongly disagree 2.4% | Participants strongly agreed with this statement, but one participant commented that service capacity may also contribute to these decisions, and another said that it may be useful to set expectations by giving typical numbers of sessions. |
| **Statement 11: A minimum number of 1 pre and 1 post-operative voice therapy sessions could be recommended as a guide for anyone undergoing phonosurgery with the option to increase this according to patient and surgical factors.**  Explanatory Statement: Patient factors may include vocal requirements, preferred learning styles, vocal skill, and level of psychosocial support. Surgical factors will include depth and complexity of surgery. | **4.17**  **(1.167)** | **83.3% Agree or Strongly Agree**  Strongly agree 52.4%  Agree 31%  Neither Agree nor disagree 2.4%  Disagree 9.5%  Strongly disagree 4.8% | Most participants strongly agreed with this statement, although there was representation across the scale. Those who strongly agreed felt it was important to see SLT as an essential part of the treatment and felt this might improve standardised patient pathways. Others wondered whether a minimum standard was sufficient. Those who disagreed, felt that minimum standards should be increased, with participants offering other minimum suggested numbers of sessions. |
