# Supplementary material for: Pre- and Postoperative Voice Therapy for Benign Vocal Fold Lesions: An International Electronic Delphi Consensus Study
Source: J Voice. 2025 May;39(3):664–75. doi: 10.1016/j.jvoice.2022.12.008 (PMC12063773; doi:10.1016/j.jvoice.2022.12.008)
Supplement: Supplementary file 4 [file mmc4.docx]

**Appendix D: Graphical representations stability of consensus between rounds**
